# Cohesin SA2 is a sequence-independent DNA-binding protein that recognizes DNA replication and repair intermediates

**DOI:** 10.1074/jbc.M117.806406

**Published:** 2020-08-27

**Authors:** Preston Countryman, Yanlin Fan, Aparna Gorthi, Hai Pan, Evelyn Strickland, Parminder Kaur, Xuechun Wang, Jiangguo Lin, Xiaoying Lei, Christian White, Changjiang You, Nicolas Wirth, Ingrid Tessmer, Jacob Piehler, Robert Riehn, Alexander J.R. Bishop, Yizhi Jane Tao, Hong Wang

**Affiliations:** a Physics Department, North Carolina State University, Raleigh, North Carolina 27695; e Biomedical Engineering Department, North Carolina State University, Raleigh, North Carolina 27695; j Center for Human Health and the Environment, North Carolina State University, Raleigh, North Carolina 27695; b Department of BioSciences, Rice University, Houston, Texas 77251; c Greehey Children's Cancer Research Institute, University of Texas Health, San Antonio, Texas 78229; d Department of Cell Systems and Anatomy, University of Texas Health, San Antonio, Texas 78229; f Institute of Biomechanics, School of Bioscience and Bioengineering, South China University of Technology, Guangzhou, Guangdong 510006, China; g School of Public Health, Shandong University, Jinan 250012, China; h Division of Biophysics, Universität Osnabrück, Barbarstrasse 11, 49076 Osnabrück, Germany; i Rudolf Virchow Center for Experimental Biomedicine, University of Würzburg, Josef-Schneider-Strasse 2, 97080 Würzburg, Germany

**Keywords:** atomic force microscopy (AFM), DNA-binding protein, fluorescence anisotropy, genomic instability, protein-DNA interaction, SA2, STAG2, cohesin DNA binding, single-molecule biophysics, fluorescence microscopy

## Abstract

Proper chromosome alignment and segregation during mitosis depend on cohesion between sister chromatids, mediated by the cohesin protein complex, which also plays crucial roles in diverse genome maintenance pathways. Current models attribute DNA binding by cohesin to entrapment of dsDNA by the cohesin ring subunits (SMC1, SMC3, and RAD21 in humans). However, the biophysical properties and activities of the fourth core cohesin subunit SA2 (STAG2) are largely unknown. Here, using single-molecule atomic force and fluorescence microscopy imaging as well as fluorescence anisotropy measurements, we established that SA2 binds to both dsDNA and ssDNA, albeit with a higher binding affinity for ssDNA. We observed that SA2 can switch between the 1D diffusing (search) mode on dsDNA and stable binding (recognition) mode at ssDNA gaps. Although SA2 does not specifically bind to centromeric or telomeric sequences, it does recognize DNA structures often associated with DNA replication and double-strand break repair, such as a double-stranded end, single-stranded overhang, flap, fork, and ssDNA gap. SA2 loss leads to a defect in homologous recombination–mediated DNA double-strand break repair. These results suggest that SA2 functions at intermediate DNA structures during DNA transactions in genome maintenance pathways. These findings have important implications for understanding the function of cohesin in these pathways.

## Introduction

In eukaryotes, proper chromosome alignment and segregation during mitosis depend on cohesion between sister chromatids (1, 2). Cohesion is mediated by the cohesin complex, which also plays important roles in diverse biological processes, including DNA double-strand break (DSB)^2^ repair, restart of stalled replication forks, and maintenance of 3D chromatin organization (3, 4). In vertebrates, cohesin consists of heterodimeric ATPases SMC1 and SMC3, a kleisin subunit RAD21 (also known as Scc1), and the stromal antigen (SA or Heat-B) subunit, which can be either SA1 (STAG1) or SA2 (STAG2). The core cohesin complex exists at 1:1:1:1 stoichiometry in cells (5). Electron microscopy–, crystallography–, and biochemical assay–based studies support the notion that cohesin binds to DNA by topological embrace through the ring subunits (SMC1, SMC3, and RAD21) (6,7, 8, 9, 10,11). SA1 and SA2 share 70% sequence homology and exist in separate cohesin complexes, with SA2 being more abundant than SA1 (12, 13,14). In addition to the core cohesin subunits, several cohesin regulatory factors have been discovered that play important roles in the loading, stability, and cleavage of the cohesin ring during different phases of the cell cycle (15,16, 17,18). Furthermore, non-SMC subunits in cohesin and condensin (Psc3, Ycg1, and Ycs4) and NSE1/3/4 from the SMC5/6 complex have been implicated in DNA binding (9, 19, 20).

Germ line mutations in core cohesin subunits or their regulators are associated with a spectrum of human diseases collectively called “cohesinopathies” and an increased incidence of cancer (3, 21, 22). Somatic mutations of the SA2 gene and loss of SA2 protein expression have been reported in multiple cancer cell lines, including urothelial bladder carcinomas, Ewing’s sarcomas, glioblastomas, and malignant melanomas (21).

Despite the progress made since the discovery of the cohesin complex, many fundamental questions regarding the structure and assembly of cohesin remain unanswered (23, 24). For example, how cohesin binds to chromatin to establish sister chromatid cohesion is not fully understood (25). Various models, including one ring, twin-ring handcuffs, bracelet oligomers, and C-clamps, have been proposed for cohesin assembly on DNA (24). However, these models have not taken into consideration that SA2 plays important roles both in stabilizing cohesin on DNA and unloading cohesin from chromatin. It is known that SA2 phosphorylation by the polo-like kinase 1 (Plk1) leads to the removal of cohesin from chromatin (26), indicating the importance of SA2 in the relationship of cohesin with DNA.

In addition, how cohesin DNA binding is spatially controlled along the genome is poorly understood. DNA DSB induction leads to the establishment of sister chromatid cohesion in the G_2_ phase, which facilitates the DNA repair process (27,28, 29, 30,31). It was proposed that following the induction of DSBs, cohesin is recruited to the region surrounding the DSB as well as genome-wide through the DNA damage response pathway and chromatin remodeling (32, 33). In addition, the *Schizosaccharomyces pombe* cohesin ring is capable of sliding on DNA with a diffusion constant approaching the theoretical limit for free 1D diffusion, and the complex falls off from free DNA ends (34). These observations raise an important question: How does the cohesin complex promote stable cohesion during DNA DSB repair without sliding off from DNA ends? In addition, SA1 and SA2 have different roles during DSB repair, as well as during sister chromatid cohesion at telomeres and centromeres (35, 36). Whereas SA2 is important for cohesion at centromeres, depletion analysis showed that telomeres relied heavily on SA1 and to a lesser extent on the cohesin ring for cohesion (35, 36).

It has been suggested that the SA subunits in humans and their orthologs in yeast (Scc3 in budding yeast and Psc3 in fission yeast) play a role in the loading of cohesin ring onto chromosomes through the interaction between DNA and the cohesin hinge (37, 38). The crystal structure of SA2 (residues 80–1060) shows that it contains a helical domain at its N terminus followed by 17 HEAT repeats shaped like a dragon (39, 40). Binding to DNA through the HEAT repeat–containing subunits has been proposed to serve as the first step in condensin loading (19). The N- and C-terminal domains of SA1 and SA2 share only 30–50% homology, which makes it likely that these domains contribute to their functional specificities. Recently, we discovered that SA1 binds to dsDNA and shows specificity for telomeric sequences (41). These new results raise an important question as to whether or not SA2 specifically recognizes unique DNA sequences or structures. Here, to investigate the binding of SA2 to specific DNA sequences and structures, we applied fluorescence anisotropy and two complementary single-molecule imaging techniques, atomic force microscopy (AFM) and fluorescence imaging of quantum dot (QD)-labeled proteins on DNA tightropes. In contrast to SA1 (41), the 1D diffusion dynamics of SA2 on DNA is independent of telomeric or centromeric sequences. Fluorescence anisotropy shows that SA2 binds to both ssDNA and dsDNA, albeit with a higher binding affinity for ssDNA. In addition, SA2 recognizes DNA overhang, flap, and fork, which are intermediate DNA structures during DNA repair, recombination, and replication. Likewise, AFM imaging reveals that SA2 displays high binding specificities for the DNA end, ssDNA gap, flap, single-stranded fork, and replication fork. Strikingly, SA2 is capable of switching between two DNA-binding modes: searching through unbiased 1D diffusion on dsDNA and recognition through stable binding at the ssDNA gap. Furthermore, results from the DR-GFP reporter system show that SA2 directly facilitates homologous recombination (HR)-mediated DNA DSB repair. Importantly, these results strongly suggest a new role for SA2 in recognizing intermediate DNA structures during genome maintenance pathways.

## Results

### SA2 specifically binds to DNA ends

**Figure 1:**
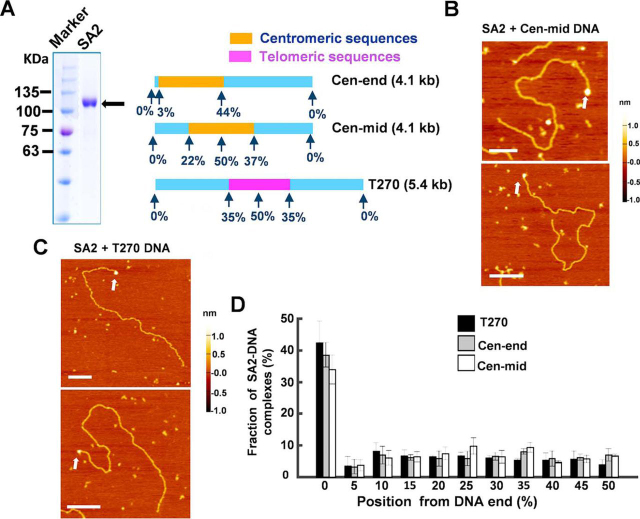
**SA2 does not show binding preference for telomeric or centromeric DNA sequences but recognizes DNA ends.***A*, SDS-polyacrylamide gel of purified full-length SA2 (*black arrow*, *left panel*) and three DNA substrates used for AFM imaging (*right panel*). *B* and *C*, representative AFM images of SA2 on the centromeric (*B*, Cen-mid) or telomeric (*C*, T270) DNA substrates. *White arrows* point to SA2 binding to DNA ends. *xy scale bars*, 200 nm. *D*, position distributions of SA2 on DNA substrates containing telomeric (T270; *n* = 283) or centromeric sequences close to one end (Cen-end; *n* = 275) or in the middle (Cen-mid; *n* = 298). *Error bars*, S.E. from at least three independent experiments.

Studying the DNA-binding properties of SA1 and SA2 is essential for advancing our understanding of the function of the cohesin complex in diverse genome maintenance pathways. Recently, we discovered that SA1 binds to DNA through the AT-hook domain at its N-terminal domain (41). SA2 lacks the AT-hook motif (36). To investigate whether or not SA2 is a DNA-binding protein, we purified His-tagged full-length SA2 (Fig. 1*A* and Methods). First, we evaluated the oligomeric state of SA2 using a previously established method that estimates the molecular mass of a protein based on the calibration curve correlating AFM volume and molecular weight of proteins (42, 43,44). Based on this method, SA2 molecules (141 kDa) display AFM volumes (146 nm^3^) consistent with being predominantly monomers (Fig. S1A). This result is consistent with our earlier analysis of SA2 molecular weight using gel filtration chromatography (45).

To evaluate SA2-DNA binding specificity, we applied AFM imaging of SA2 in the presence of linear DNA fragments containing either centromeric or telomeric sequences (Fig. 1*A* and Methods). Ensemble-based biochemical assays, such as fluorescence anisotropy and EMSAs, only provide average binding affinities for DNA substrates. These assays cannot differentiate sequence-specific DNA binding from DNA end binding. In contrast, from AFM images of protein-DNA complexes, a direct measurement of the DNA-binding specificity for unique sequences as well as that for DNA structures such as ends can be obtained through statistical analysis of binding positions of protein complexes on individual DNA fragments (46). Two centromeric DNA substrates (4.1 kb) used for AFM imaging contain the α-satellite centromeric sequences that are either close to one end of the linearized DNA (Cen-end DNA) or near the middle (Cen-mid DNA) (Fig. 1*A*). For the telomeric DNA substrate (T270 DNA), the (TTAGGG)_270_ sequences make up ∼30% of the total DNA length (5.4 kb) and are located at the middle of the linearized T270 DNA (Fig. 1*A*). SA2 molecules displayed AFM heights (1.41 ± 0.30 nm, mean ± S.D., Fig. 1 (*B* and *C*) and supplemental Fig. S1B) that were significantly taller than that of dsDNA alone (0.70 ± 0.08 nm, mean ± S.D.). This large difference in heights enabled unambiguous identification of SA2 molecules on DNA. Statistical analysis of the binding position of SA2 on DNA revealed that SA2 did not bind specifically to either the centromeric or telomeric sequences (Fig. 1*D*). However, on all three DNA substrates, the majority of SA2 molecules were bound at the DNA ends. Furthermore, DNA end binding by SA2 was independent of the internal DNA sequence, the position of the centromeric region, or the presence of single-stranded overhangs at the terminal ends (4-nt 3′-overhang on Cen-end DNA; Fig. 1*D*).

To further quantify the SA2-binding specificity for DNA ends, we applied the analysis based on the fractional occupancies of SA2 at DNA ends (46). SA2-binding specificities for DNA ends (*S* = DNA binding constant for specific sites/DNA binding constant for nonspecific sites = *K*_SP_/*K*_NSP_) are 2945 ± 77, 2604 ± 68, and 2129 ± 76, respectively, for T270, Cen-end, and Cen-mid DNA substrates. In addition, in contrast to SA2 alone, DNA-bound SA2 formed higher-order oligomeric complexes with average AFM volumes of 1025 ± 88 and 898 ± 63 nm^3^, respectively, at DNA ends and internal sites (Fig. S1C). Based on the calibration curve relating protein molecular weights and AFM volumes (44), these AFM volumes correspond to approximately five and four SA2 molecules, respectively, at the DNA ends and internal sites. In summary, SA2 does not specifically bind to centromeric sequences, but binds DNA ends with high specificities that are independent of DNA sequences and short (4-nt) single-stranded overhangs.

### SA2 binds to the ssDNA gap with high specificities

**Figure 2:**
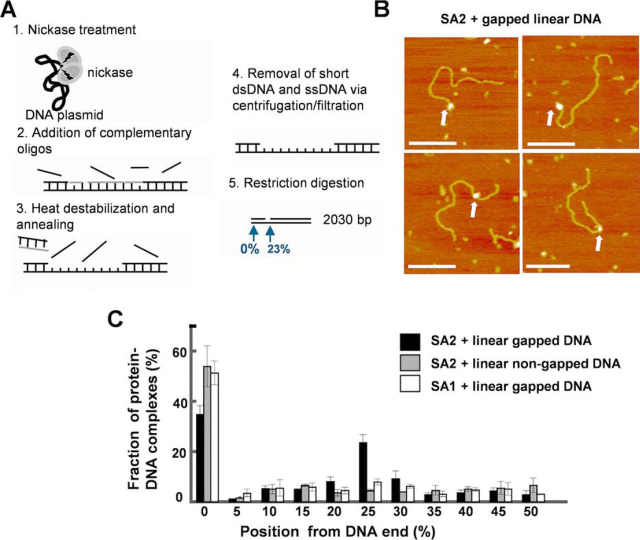
**SA2 specifically binds to ssDNA gaps.***A*, generation of the linear gapped DNA substrate. Gapped DNA was created using pUC19-derived pSCW01 plasmid (duplication of 2030 bp) that contains closely spaced Nt.BstNBI nicking sites. After restriction digestion, the resulting ssDNA gap (37 nt) is located at 470 nt (23%) from one end of the linear DNA fragment (2030 bp). *B*, representative AFM images of the full-length SA2 complex binding to the linear gapped DNA substrate. The contour length of the linear gapped DNA was measured as *L*_c_ = 622.5 ± 41.3 nm. *xy scale bars*, 200 nm. *C*, statistical analysis of the position distribution of the full-length SA2 complex on the linear gapped (*n* = 251) and non-gapped (*n* = 201) DNA as well as the full-length SA1 on the gapped DNA substrate (*n* = 295). The position of SA2 was measured from the closest DNA end (0–50%). Consequently, there are two binding sites for DNA ends and one binding site for ssDNA gap. Each data set was obtained from at least two independent experiments. *Error bars*, S.E.

Previously, it was established that cohesin deposition and establishment occur in concert with lagging strand-processing (47). ssDNA gaps are intermediate structures on lagging strand during DNA replication. To directly test whether or not SA2 binds to ssDNA gaps, we used a previously established method to generate a linear substrate containing an ssDNA gap (37 nt) flanked by dsDNA arms (Fig. 2*A*). This method was based on the generation of four closely spaced nicks using DNA nickase and subsequent removal of short ssDNA between nicked sites using complementary oligonucleotides (48, 49). After restriction digestion of the circular gapped DNA, the ssDNA gap is at 470 nt (23%) from one end of the DNA (blunt end; Fig. 2*A* and Fig. S2A). Based on diagnostic restriction digestion at the gapped region, DNA gapping efficiencies were typically 85–95% (Fig. S2B). To further confirm the presence of the ssDNA gap, the position distribution of mitochondrial single-stranded DNA-binding protein on this DNA substrate was analyzed. Mitochondrial single-stranded DNA-binding protein predominantly bound to the expected ssDNA region on the gapped DNA substrate, whereas its binding on the nicked DNA substrate was random.^3^ In summary, these results established the presence of a ssDNA gap at the defined location on the linear gapped DNA substrate.

Next, to study whether or not SA2 specifically binds to ssDNA gaps, we directly compared SA2 binding on non-gapped (without nickase treatment) with binding on gapped DNA substrates (Fig. 2, *B* and *C*). AFM imaging showed that on the non-gapped DNA substrate, SA2, predominantly bound to the DNA ends, and its distribution at internal sites along the linear DNA fragment was random (Fig. 2*C*). This is consistent with position distributions of SA2 on telomeric and centromeric DNA substrates (Fig. 1*D*). In stark contrast, the presence of an ssDNA gap shifted the SA2 binding from the DNA end to a region consistent with the location of the ssDNA gap (23% along the length of the DNA; Fig. 2*C*). Analysis of the fractional occupancies of SA2 on DNA demonstrated that SA2 displayed high binding specificities (*S* = 1994 ± 54) for the ssDNA gap. In addition, compared with the size of SA2 molecules positioned outside the gapped regions (1096 ± 117 nm^3^), at the ssDNA gaps, SA2 formed larger complexes with a broader size distribution (1458 ± 232 nm^3^; Fig. S1C).

Because DNA nicking is the intermediate step for generating DNA gaps, we further tested whether or not SA2 specifically binds to DNA nicks. First, to evaluate whether SA2 displays binding specificities for individual nick sites, we generated a third DNA substrate that is a linear DNA substrate (517 bp) containing a single nick site at 37% from one DNA end (50). DNA nicking was confirmed by the observation of slower mobility of nicked DNA in comparison with its non-nicked counterpart under gel electrophoresis (Fig. S3A). On the nicked DNA substrate, SA2 displayed preferential binding to DNA ends (Fig. S3A). In stark contrast to what was observed on the gapped DNA substrate, along the nicked DNA substrate, SA2 molecules were randomly distributed at internal sites (Fig. S3A). Furthermore, on a DNA substrate containing five nick sites spatially separated from one another, AFM imaging further established that SA2 did not show a preference for nicked sites (Fig. S3B). In addition, a previous study showed that the C terminus of SA2 confers DNA damage site–targeting specificity on SA1 (51). To further understand SA2 DNA binding, we investigated whether SA2 with C-terminal domain deletion retains DNA-binding properties. AFM imaging showed that SA2 1–1051 retains DNA-binding specificities for DNA ends (S = 1687 ± 82) and ssDNA gaps (*S* = 1813 ± 79; Fig. S4). In contrast, AFM imaging showed that SA1 also displays high binding specificity for DNA ends (*S* = 2094 ± 38), but not for the 37-nt ssDNA gap (Fig. 2*C*) or nick sites (Fig. S5). In summary, these results show that SA2 displays high binding specificities for ssDNA gaps, but not DNA nicks. SA2 with C-terminal domain deletion retains binding specificities for DNA ends and ssDNA gaps.

### SA2 carries out sequence-independent unbiased 1D diffusion on dsDNA

**Figure 3:**
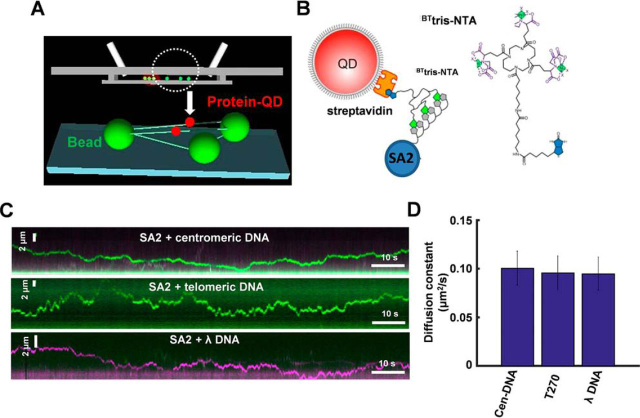
**SA2 displays similar binding dynamics on DNA substrates containing centromeric, telomeric, or random sequences.***A*, schematic of the DNA tightrope assay. *Green* and *red spheres* represent poly-l-lysine–treated silica beads and red QD-labeled protein molecules, respectively. *B*, QD conjugation strategy: a His-NTA-biotin-QD sandwich method for conjugating His-tagged SA2 to QDs using ^BT^tris-NTA as the linker. *C*, representative kymographs of QD-labeled SA2 on centromeric (*top*), telomeric (*middle*), and λ DNA tightropes (*bottom*). In all reactions, SA2 protein was incubated with both *red* (655 nm) and *green* (565 nm) QDs at equal molar concentrations. *D*, diffusion constants of SA2 on centromeric (Cen-DNA; *D* = 0.10 ± 0.02 μm^2^ s^−1^, *n* = 48), telomeric (T270; *D* = 0.10 ± 0.02 μm^2^ s^−1^, *n* = 53), or λ (*D* = 0.09 ± 0.02 μm^2^ s^−1^, *n* = 48) DNA tightropes. *Error bars*, S.E.

Target search through 3D diffusion and/or dynamic movements on DNA, such as 1D sliding, jumping, and hopping, are essential for proteins to find their recognition sites on DNA (52,53, 54,55). To understand how proteins dynamically achieve DNA-binding specificities, we developed a DNA tightrope assay based on oblique angle total internal reflection fluorescence microscopy imaging of QD-labeled proteins on DNA stretched between 1-μm-sized silica beads (41, 56,57, 58,59). DNA tightropes (at an elongation of ∼90% of the contour length) are formed between poly-l-lysine–treated silica microspheres using hydrodynamic flow (Fig. 3*A*) (57). To generate longer DNA substrates with specific sequences that can span between silica microspheres, we ligated linear DNA fragments containing genomic, telomeric, or centromeric DNA sequences (Fig. 1*A*) (57). Recently, using the DNA tightrope assay, we observed that QD-labeled SA1 displays slow subdiffusive events amid fast unbiased 1D diffusion in a telomeric sequence-dependent manner (41).

To study SA2-DNA binding dynamics, the streptavidin-coated QD was conjugated to His-SA2 using biotinylated multivalent chelator tris-nitrilotriacetic acid (^BT^tris-NTA) as the linker (Fig. 3*B*) (60). The three Ni^2+^-NTA moieties on the circular scaffold of the tris-NTA adaptor bind to a His tag with subnanomolar affinities (60). AFM imaging revealed that QDs in the presence of only ^BT^tris-NTA did not have significant binding affinities for DNA. Under the conditions used in this study (SA2/QD = 4:1), AFM imaging showed that the majority (87%) of the SA2-QD conjugates displayed a single SA2 molecule attached to individual QDs (Fig. S6). The addition of His-tagged SA2 to the ^BT^tris-NTA-QD reaction led to the loading of QDs onto DNA, indicating that QD binding to DNA tightropes was mediated through SA2. In addition, SA2-QDs retained DNA-binding specificities toward ssDNA gaps (Fig. S7). To monitor SA2 binding on DNA in real time, QD-labeled SA2 molecules were introduced into the flow cell using a syringe pump after DNA tightropes were established between poly-l-lysine–treated silica microspheres. Then the flow was stopped, allowing freely diffusing SA2 molecules in solution to bind to DNA tightropes (Fig. 3*C* and Movie S1). On all DNA substrates, SA2-QD molecules on DNA were long-lived, with ∼80% of SA2-QD complexes remaining on DNA tightropes after 2 min (*n* = 277). The positions of SA2-QDs were tracked by Gaussian fitting to intensity profiles to obtain the diffusion constant (41, 56, 57). Importantly, at the same protein concentrations (5 nm in the flow cell), the diffusion constants of SA2 on λ DNA and DNA tightropes containing either telomeric or centromeric sequences are indistinguishable (Fig. 3*D* and Table S1). In addition, the α-factor (diffusive exponent) was calculated to determine whether SA2 displayed subdiffusive motion on DNA. An α-factor of 1 indicates an unbiased random walk, and a value of <1 indicates periods of pausing in the random walk process (subdiffusion) (61). Recently, we found that SA1 shows telomeric sequence–dependent subdiffusive behavior on DNA, manifested by an α-factor significantly smaller than 1 (α-factor = 0.69 ± 0.03 on telomeric DNA) (41). SA2 displayed free 1D diffusion on centromeric DNA (α-factor = 0.96 ± 0.02) and λ DNA (α-factor = 0.93 ± 0.04) tightropes (Table S1). In comparison, the α-factors displayed by SA2 on telomeric DNA tightropes were only slightly (*p* = 0.01) lower (0.86 ± 0.03). In summary, fluorescence imaging of QD-labeled SA2 on DNA tightropes directly shows that SA2 carries out sequence-independent 1D diffusion on DNA tightropes containing telomeric, centromeric, or genomic sequences. These results are consistent with random position distributions of SA2 on both telomeric and centromeric DNA substrates shown in AFM images (Fig. 1*D*).

### SA2 switches between dsDNA and ssDNA gap-binding modes

**Figure 4:**
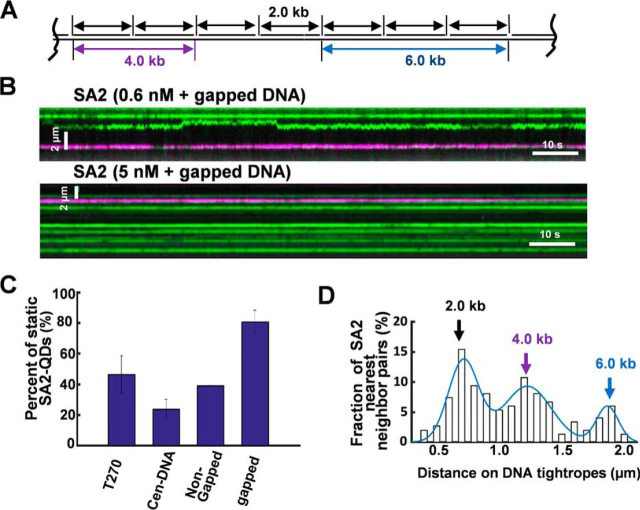
**SA2 stably binds to ssDNA gaps.***A*, schematic of the DNA tightropes with 37-nt ssDNA gaps at defined spacing. *B*, representative kymographs of SA2 on the ligated DNA tightropes containing gaps at the low (0.6 nm) and standard (5 nm) protein concentrations. The ssDNA gaps were generated by heating and introduction of complementary oligonucleotides after the DNA tightropes were formed. Equal molar concentrations of red and green QDs were present in the conjugation reactions. *C*, percentage of static SA2 molecules on telomeric (46 ± 12%, *n* = 121 total), centromeric (24 ± 6%, *n* = 156 total), non-gapped control (39%, *n* = 79 total), and gapped (81 ± 19%, *n* = 166 total) DNA tightropes. The final SA2 concentration in the flow cell was 0.6 nm. *D*, statistical analysis of the spacing between SA2-QD complexes on the gapped DNA tightropes (*n* = 149). The *line* represents the Gaussian fit to the data (*R*^2^ > 0.93) with peaks centered at 0.72 (∼2.0 kb), 1.23 (∼4.0 kb), and 1.87 (∼6.0 kb) μm, respectively. *Error bars*, S.E.

To study SA2 DNA-binding dynamics on DNA tightropes containing gaps, we introduced ssDNA gaps after anchoring ligated DNA between silica microspheres (Fig. 4*A*). Generation of ssDNA gaps on DNA tightropes was carried out by introducing the nickase and complementary oligonucleotides in the flow cell, followed by heating it at 55 °C and washing with high-salt buffers to remove nickase and excess short ssDNA and dsDNA (Fig. 2*A*). Restriction digestion confirmed the presence of ssDNA gaps on DNA tightropes. YOYO1-stained non-gapped DNA tightropes between silica microspheres disappeared after treatment with three restriction enzymes targeting the sequences between the nickase recognition sites. In contrast, the gapped DNA tightropes stayed intact. These observations confirmed the establishment of ssDNA gaps on DNA tightropes. Compared with SA2 on telomeric (46%), centromeric (24%), and non-gapped control DNA (39%) on DNA tightropes containing ssDNA gaps, a significantly (*p* < 10^−6^) higher percentage of SA2 molecules were static (81%; Fig. 4 (*B* and *C*) and Table S1). In addition, the density of SA2 on gapped DNA tightropes increased with higher SA2 concentrations (Fig. 4*B*, compare *top* and *bottom*). To evaluate whether or not the static SA2-binding events occurred at the gapped region, we measured the distance between nearest neighbor SA2-QD pairs. The distribution of this distance shows three distinct peaks centered at 0.72, 1.23, and 1.87 μm, respectively (Fig. 4*D*), which are consistent with the expected spacing between ssDNA gaps on the ligated DNA tightropes (Fig. 4*A*). In stark contrast, on DNA tightropes containing nicks, the spacing between nearest neighbor SA2-QD pairs was random (Fig. S3C).

**Figure 5:**
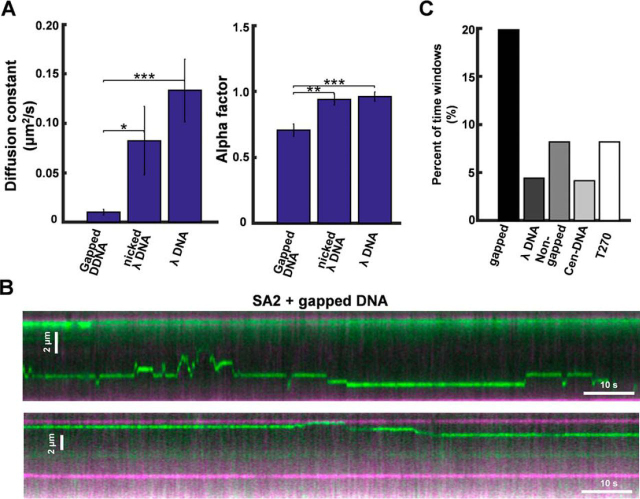
**SA2 switches between searching and recognition modes on DNA tightropes containing ssDNA gaps.***A*, comparison of SA2 diffusion constants and α-factors on gapped DNA (*n* = 28), nicked λ DNA (*n* = 20), and non-nicked λ (*n* = 20) DNA (Table S1). Final SA2 concentration was 0.6 nm in the flow cell. *, *p* < 0.02; **, *p* < 0.001; ***, *p* < 0.0005. *B*, kymographs of SA2 showing individual SA2 molecules alternating between 1D diffusion and stable binding on gapped DNA tightropes. *C*, percentages of time windows (40 frames/2 s) with *D*_int_ values less than 1.0 × 10^−4^ for mobile SA2 on gapped, λ, non-gapped control, centromeric (Cen-DNA), and telomeric (T270) DNA tightropes. Histograms of *D*_int_ are shown in Fig. S8. *Error bars*, S.E.

To further confirm that DNA-binding dynamics of SA2 on gapped DNA tightropes is distinctly different from that on nicked DNA, we compared the diffusion constant and α-factor of mobile SA2 on DNA containing ssDNA gaps and λ DNA (untreated or nicked) tightropes (Fig. 5*A*). We introduced nicked sites by incubating λ DNA with Nt.BstNBI nickase. To remove nickase, nicked λ DNA was further purified using phenol chloroform extraction before being introduced into the flow cell. λ DNA has >40 Nt.BstNBI nickase sites, with spatial separation ranging from 13 to >2000 bp. To observe mobile SA2 complexes on DNA tightropes, the final SA2-QD concentration in the flow cell (0.6 nm) was kept the same across all DNA substrates but lower than the standard concentration (5 nm; Fig. 3 and Table S1). On gapped DNA tightropes, SA2 showed a significant (*p* < 0.02) decrease in the diffusion constant and α-factor (*D* = 0.01 ± 0.003 μm^2^ s^−1^ and α-factor = 0.70 ± 0.05) compared with untreated λ (*D* = 0.13 ± 0.03 μm^2^ s^−1^ and α-factor = 0.96 ± 0.03) or nicked λ DNA tightropes (*D* = 0.08 ± 0.03 μm^2^ s^−1^ and α-factor = 0.94 ± 0.04; Fig. 5*A*).

Interestingly, on the gapped DNA tightropes, a subpopulation of mobile SA2 molecules (*n* = 21 of 150) alternated between mobile and static binding modes (Fig. 5*B* and Movie S2). These apparent static binding events could be due to SA2 binding or sliding within a narrow range below the resolution of our imaging platform (16 nm after Gaussian fitting) (57). The pairwise distance between nearest neighbor static SA2-binding positions was 0.60 ± 0.19 μm (*n* = 21), which is consistent with the spacing between two adjacent ssDNA gaps (2.0 kb) on DNA tightropes (Fig. 4*A*). To further compare SA2 DNA-binding dynamics on different DNA substrates, we calculated a time interval-based diffusion constant (*D*_int_, Fig. S8) by mobile SA2 using a “sliding window” (40-frame, 2 s) mean square displacement (MSD) analysis (41). This analysis indicated that distinct from the unbiased 1D diffusion mode (*D*_int_ ∼1.0 × 10^−2^ μm^2^ s^−1^) on the centromeric (Fig. S8A), telomeric (Fig. S8B), and λ DNA (Fig. S8C), mobile SA2 molecules displayed an additional population with *D*_int_ values centered at ∼1.0 × 10^−4^ μm^2^ s^−1^ on gapped DNA tightropes (Fig. S8D). Furthermore, we used a *D*_int_ value of 1.0 × 10^−4^ μm^2^ s^−1^ as the threshold value to identify individual static binding events. This value is based on the nominal diffusion constant values measured from static protein-QDs on DNA tightropes (41). This analysis indicated that on the gapped DNA tightropes, mobile SA2 molecules displayed a significantly (*p* = 0.002) higher percentage (∼20%) of time windows (40-frame, 2 s) in the static binding mode (Fig. 5*C*) compared with other DNA substrates (<8% for telomeric, centromeric, λ, and non-gapped control).

Taken together, fluorescence imaging of QD-labeled SA2 establishes that SA2 alternates between two DNA-binding modes on gapped DNA: unbiased 1D diffusion on dsDNA (search mode) and stable binding (recognition mode) at ssDNA gaps.

### SA2 forms higher-order oligomeric complexes and can bypass diffusion barriers on DNA

In AFM images, whereas SA2 alone mainly existed as monomers, SA2 formed higher-order oligomers on DNA (Fig. S1C). Consistent with these observations using AFM, SA2-QDs with brighter intensities were observed to break up into multiple fainter ones (Fig. S9A, yellow arrows). This observation indicated that the brighter SA2 complexes were higher-order oligomers. To determine how SA2 dynamically forms higher-order oligomeric complexes on DNA, we analyzed instances where a mobile SA2 molecule encountered additional stationary or mobile SA2 molecules. The overwhelming majority (92%, *n* = 49) of SA2-SA2 interactions on DNA were collisions that did not form complexes. However, there were cases (8%) of initial separate mobile SA2 molecules that collided and then diffused in synchronicity with brighter intensity than individual molecules (Fig. S9A, white arrows). The diffusion constant of larger oligomers of SA2 on DNA tightropes (*n* = 9 complexes on centromeric, telomeric, and gapped DNA) is 0.01 ± 0.02 μm^2^ s^−1^, which is ∼10 times slower than individual SA2 complexes observed in the DNA tightrope assay. For SA2 oligomers, only 7.6% of the time windows (*n* = 3079) shows *D*_int_ values <1.0 × 10^−4^ μm^2^ s^−1^, which is consistent with the α-factor (0.92 ± 0.04) and suggests that these higher-order oligomers of SA2 carried out unbiased 1D diffusion without significant pausing events. Combined with the observation that SA2 by itself mainly exists in the monomeric form (Fig. S1A), these results imply that SA2 binds directly to DNA as monomers from the solution; the assembly of higher-order SA2 complexes on DNA is promoted through 1D diffusion and direct interactions between SA2 molecules on DNA.

Proteins that maintain continuous close contact with DNA during sliding are unable to circumnavigate obstacles posed by another protein on DNA. In contrast, a hopping mechanism in which a protein microdissociates and reassociates with DNA within a distance comparable with or greater than the dimension of DNA-bound proteins could enable it to transverse these diffusion barriers. Previously, single-molecule imaging has revealed hopping by a DNA repair protein (Mlh1-Pms1) and p53 (62, 63). We observed instances of mobile SA2 molecules (*n* = 4 of 49 colliding SA2 pairs) bypassing another DNA-bound SA2 molecule (Fig. S9B and Movie S3). This bypass frequency is comparable with what was observed with Mlh1-Pms1 (62).

### SA2 binds to DNA intermediate structures associated with DNA repair and replication

**Figure 6:**
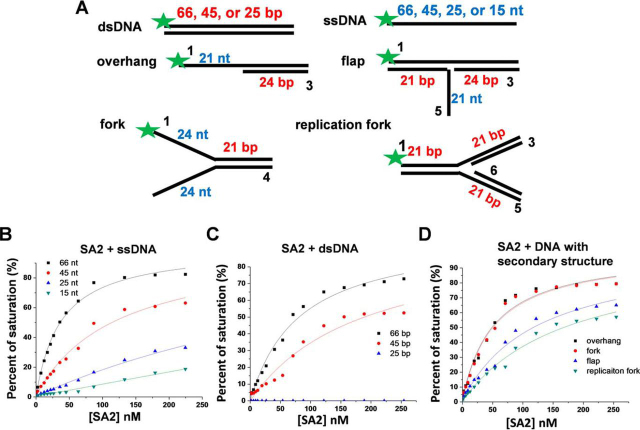
**SA2 binds to both dsDNA and ssDNA substrates and shows preference for DNA substrates mimicking intermediate structures during DNA recombination, repair, and replication.***A*, schematic illustration of DNA substrates used for fluorescence anisotropy experiments with *numbers* in *black* correlating with the sequences outlined in Fig. S10A. The numbers in nt and bp denote the lengths of the ssDNA and dsDNA regions, respectively, on the DNA substrates. The *green star* represents the fluorescent dye (Alexa 488). *B–D*, fluorescence anisotropy experiments showing concentration-dependent binding of SA2 to ssDNA (*B*; 15, 25, 45, and 66 nt), dsDNA (*C*; 25, 45, and 66 bp), and DNA with secondary structures (*D*; overhang, flap, fork, and replication fork). The data were fitted to the law of mass action. The equilibrium dissociation constants are summarized in Table S2.

To further investigate DNA structures that SA2 recognizes, we next used a fluorescence anisotropy assay and compared SA2 binding to ssDNA (66, 45, and 25 nt) and dsDNA (66, 45, 25, and 15 bp) of different lengths (Fig. 6*A* and Fig. S10). These experiments showed that SA2 binds to double- and single-stranded DNA substrates in a length-dependent manner (Fig. 6 (*B* and *C*) and Table S2). There was no detectable SA2 binding for 25-bp DNA, indicating that the binding site size of SA2 on dsDNA is >25 bp (Fig. 6*C*). Importantly, for all dsDNA and ssDNA substrates tested, SA2 displays consistently higher binding affinities for ssDNA (66, 45, and 25 nt) than for dsDNA at the same length (Table S2). In addition, SA2 DNA-binding affinity for telomeric sequences (*K_d_* = 88.0 ± 1.5 nm) is comparable with that for non-telomeric DNA (*K_d_* = 76.2 ± 3.9 nm and Table S2).

Previous studies have demonstrated the role of the cohesin complex in DNA recombination and restart of DNA replication after fork stalling (64, 65). Therefore, we investigated a series of DNA substrates (overhang, flap, fork, and replication fork) that mimic DNA recombination, repair, and replication intermediates (Fig. 6 (*A* and *D*) and Fig. S10) (20). SA2 shows higher DNA-binding affinities for DNA substrates with secondary structures, including overhang (*K_d_* = 56.4 ± 9.0 nm), flap (*K_d_* = 103.8 ± 11.7 nm), fork (*K_d_* = 58.4 ± 9.4 nm), and replication fork (*K_d_* = 132.7 ± 33.5 nm) substrates than for dsDNA (*K_d_* = 175.3 ± 12.9 nm) of the same length (Fig. 6*D* and Table S2). It is worth noting that among four DNA substrates with secondary structures, SA2 displays higher binding affinities for overhang and fork DNA substrates that contain double- and single-stranded junctions (Fig. 6*D* and Table S2). With C-terminal domain deletion, SA2 1–1051 retains DNA-binding affinities, with *K_d_* of 28.0 and 198.2 nm for ssDNA (66 nt) and dsDNA (66 bp), respectively (Fig. S11, A and B). In comparison, SA1 also displayed ssDNA-binding affinities with *K_d_* of 36.5 nm (66 nt; Fig. S11C).

**Figure 7:**
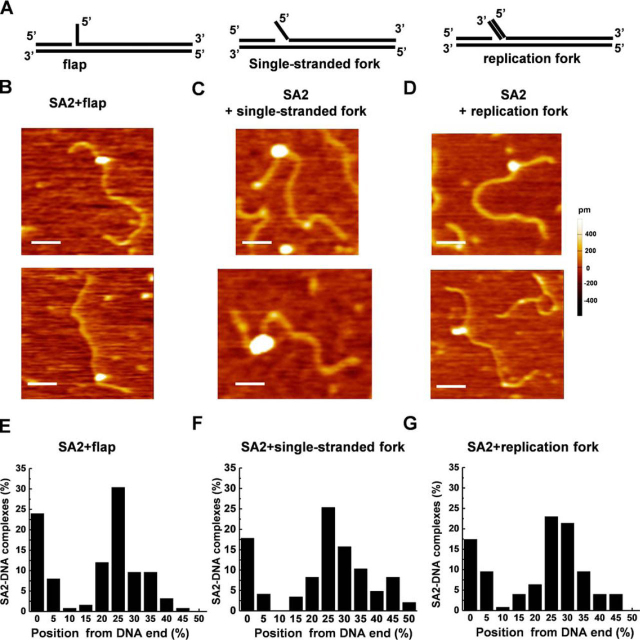
**Full-length SA2 recognizes flap, fork, and replication fork structures on linear dsDNA.***A*, *schematic models* of the linear dsDNA substrates containing flap (*left*), single-stranded fork (*middle*), and replication fork (*right*) structures at 23% from one DNA end. The substrates were generated by filling the 37-nt ssDNA region with different oligonucleotides with sequences shown in Fig. S10C. Validation of the DNA substrates is shown in Fig. S2C. *B–D*, representative AFM images of the full-length SA2 complexes binding to linear DNA substrates containing flap (*B*), single-stranded fork (*C*), and replication fork (*D*). *xy scale bars*, 100 nm. *E–G*, statistical analysis of the position distribution of the full-length SA2 complex on the linear DNA containing flap (*E*; *n* = 125), single-stranded fork (*F*; *n* = 146) DNA, and replication fork (*G*; *n* = 126). The position of SA2 was measured from the closest DNA end (0–50%). Each data set was obtained from two independent experiments.

To investigate whether or not SA2 binds to DNA substrates with secondary structures in the context of long linear dsDNA, we generated DNA substrates containing a flap, single-stranded fork, or replication fork by filling in the 37-nt ssDNA gap region (Fig. 2*A*) with unique oligonucleotides (Fig. 7*A* and Fig. S10C). The success of the annealing of additional oligonucleotides to the gapped region and formation of the dsDNA tail on the replication fork substrate were validated using restriction digestion and by monitoring the fluorescence signal from the second oligonucleotide duplexed to the ssDNA fork (Fig. S2C). Incubation of SA2 with flap, single-stranded fork, or replication fork DNA substrates led to SA2-DNA complexes with heights (1.5 ± 0.9 nm, *n* = 295; Fig. 7 (*B–D*)) that were significantly (*p* < 0.05) greater than DNA alone. Analysis of the fractional occupancies of SA2 on the linear flap, single-stranded fork, and replication fork DNA substrates in AFM images demonstrated that for all three substrates (Fig. 7, *E–G*), SA2 displayed binding specificities (*S* = 4261 for flap, *S* = 2950 for single-stranded fork, and *S* = 3147 for replication fork) that were higher than what was observed for the ssDNA gap (*S* = 1994).

**Figure 8:**
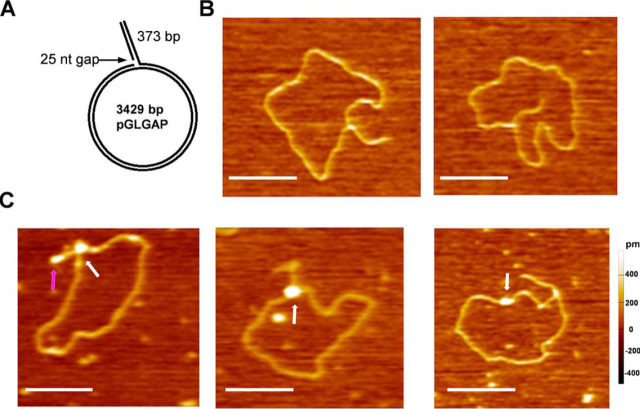
**Visualization of the full-length SA2 binding to the junction at the DNA replication fork structure.***A*, *schematic model* of the replication fork template containing a duplex circle (3429 bp) with a dsDNA tail (373 bp) and a 25-nt ssDNA gap at the junction of the circle and the tail. The DNA replication fork was created by first generating a 398-nt ssDNA tail using the Klenow fragment in the absence of dCTP after nicking, followed by conversion of the ssDNA tail to a dsDNA tail through extension of an annealed oligonucleotide. *B*, representative AFM images of the circular DNA replication fork substrate. *C*, representative AFM images of SA2 binding to the junction at the DNA replication fork (*white arrows*, *left* and *middle panels*), at the end of the dsDNA tail (*purple arrow*, *left panel*), and along the circular dsDNA (*white arrow*, *right panel*). *xy scale bars*, 200 nm.

**Figure 9:**
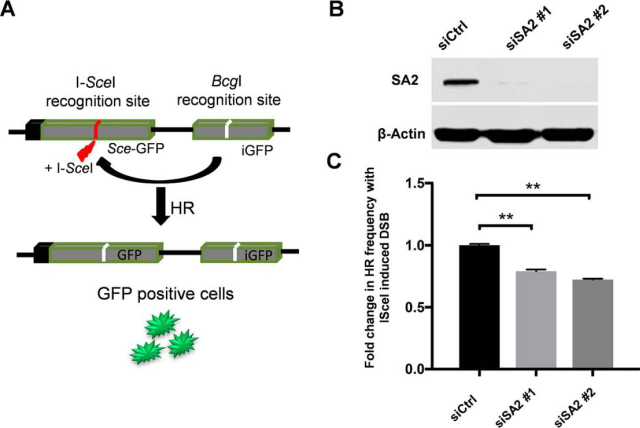
**Knockdown of SA2 leads to decreased frequencies of HR-mediated DNA DSB repair.***A*, schematic of the DR-GFP construct integrated into U2OS cells. The construct contains two tandem repeats of non-functional GFP gene interrupted by a puro cassette. The upstream GFP is rendered non-functional by replacing the BcgI site with the I-SceI restriction enzyme recognition site (Sce-GFP). The downstream repeat is an internal fragment (iGFP) containing the BcgI site. I-SceI enzyme expression results in a double-strand break at the recognition site, which, if repaired by HR using the downstream template, results in functional GFP gene expression. The figure is adapted from Gunn *et al.* (69). *B*, immunoblot showing knockdown of SA2 by two independent siRNAs in DR-GFP U2OS cells. *C*, bar graph demonstrating significant decrease in HR frequency upon loss of SA2 in DR-GFP U2OS cells 96 h after being transfected with the I-SceI plasmid. Data are represented as -fold change in frequency of HR events over baseline (siCtrl). The results (mean ± S.D. (*error bars*)) are from three independent experiments (30,000 live cells/sample). **, *p* < 0.005.

To further investigate whether or not SA2 directly binds to the junction at a DNA replication fork, we created a replication fork template containing a duplex circle (3429 bp) with a dsDNA tail (373 bp) and a 25-nt ssDNA gap at the junction of the circle and the tail (Fig. 8*A*). The replication fork template was created by generating an ssDNA tail through nick translation using the Klenow fragment over a 398-bp G-less cassette in the absence of dCTP. A dsDNA tail was then created by annealing an oligonucleotide to the ssDNA tail and strand extension by the Klenow fragment. Analysis of AFM images of the circular replication fork substrate showed that 80% of the circular DNA molecules contain dsDNA tails with the expected length (129.5 ± 19.6 nm, *n* = 45; Fig. 8*B*), which corresponds to ∼400 bp, assuming 0.32 nm/bp. Upon incubation of SA2 with the circular DNA replication template, AFM imaging revealed that 26% of the circular replication DNA molecules (*n* = 242) were bound by SA2 complexes with heights (1.6 ± 0.9 nm) significantly greater than DNA alone. Furthermore, the majority of SA2 molecules (55.6%) bound at the junction of the replication fork (Fig. 9*C*), whereas the rest of SA2 complexes bound either at the end of the dsDNA tail (12.7%) or along the circular dsDNA region (31.7%). These results are comparable with what was observed for p53, WRN helicase, and the UL8 subunit from the herpes simplex virus replication machinery on the same DNA substrate (66, 67).

Taken together, these results clearly establish that SA2 binds to both ssDNA and dsDNA in a length-dependent manner, albeit with a higher binding affinity for ssDNA than for dsDNA. SA2 does not specifically bind to telomeric sequences. Importantly, both fluorescence anisotropy and AFM imaging established that SA2 preferentially binds to DNA substrates mimicking intermediate structures that occur during DNA recombination, repair, and replication (68).

### Knockdown of SA2 leads to decreased frequency of HR-mediated DNA DSB repair

The observation of binding by SA2 to DNA with secondary structures, such as longer single-stranded DNA overhangs and single-stranded DNA flaps, raises the possibility that SA2 plays a direct role in genome maintenance pathways, such as DNA DSB repair. However, previous studies of cohesin function in these pathways were carried out only in the context of knocking down the cohesin ring subunits or by measuring the frequency of sister chromatid exchange (51, 65). Based on the observation that SA2 has higher affinity for ssDNA than dsDNA and binds to DNA with secondary structures that could be intermediates of HR, we examined whether SA2 is involved in HR-mediated repair of DNA DSB using a previously established DR-GFP reporter assay (Fig. 9*A*) (69). DR-GFP U2OS cells used in this study contain a single chromosomally integrated copy of the DR-GFP reporter. DR-GFP consists of two differentially mutated *GFP* genes (Sce-GFP and iGFP) oriented as direct repeats and separated by a drug selection marker (Fig. 9*A*). Transfection of I-SceI endonuclease introduces a DSB in Sce-GFP. Homologous recombination through non-crossover short-tract gene conversion (the majority of HR events in mammalian cells for the DR-GFP reporter system) using the downstream iGFP repeat as the repair template restores a functional GFP that can be detected by flow cytometry. The DR-GFP reporter assay has a sensitivity for detecting recombinants at a level of 10^−4^ or less (70). I-SceI expression in DR-GFP U2OS cells after transfection of a control siRNA led to 5.8 ± 0.35% of cells being GFP-positive, which is consistent with previous studies (71). Importantly, with I-SceI–induced DNA DSBs, knocking down SA2 using siRNA with the same sequence as what was used in previous studies (35, 36, 72) (Fig. 9*B*) significantly (*p* < 0.005) reduced the HR frequency to ∼75% of what was observed with control siRNA (Fig. 9*C*). In summary, these results directly demonstrate that SA2 facilitates HR-mediated DNA DSB repair.

## Discussion

Despite the importance of SA2 in multiple genome maintenance pathways, the mechanisms underlying the function of SA2 had been elusive. In this study, we establish that SA2 preferentially binds to DNA ends and DNA substrates, mimicking the intermediate structures during DNA recombination, repair, and replication. These findings have important implications for understanding the function of cohesin in diverse genome maintenance pathways.

It is well established that SA1 and SA2 have overlapping as well as unique functions (35, 73, 74). Cohesin SA1 plays a more prominent role than SA2 in the regulation of gene expression (74). One major structural difference between SA1 and SA2 proteins is found in the first 75 amino acids of their N-terminal domains (36). Only SA1 contains an AT-hook motif at its N-terminal domain. In a recent study, we propose a model in which SA1 is the “DNA sequence guide” (using its AT-hook motif) and directs the loading of the core cohesin complex at AT-rich DNA sequences along the genome (41). In this study, fluorescence anisotropy experiments demonstrate that SA2 displays higher binding affinities for ssDNA compared with dsDNA. On the other hand, SA1 displays comparable DNA-binding affinities for double-stranded telomeric DNA (*K_d_* = 34.0 ± 5.8 nm) (41) and ssDNA (*K_d_* = 36.5 ± 0.2 nm). Therefore, the specificity of SA1 for ssDNA gaps can be masked by its preference for AT-rich sequences. The overlapping DNA-binding properties between SA1 and SA2 that we demonstrate in this study are consistent with a recent report of synthetic lethality between SA1 and SA2 across different cancer contexts (75).

In this study, we discovered that unlike SA1 (41), SA2 does not specifically recognize either telomeric or centromeric DNA sequences. However, strikingly, fluorescence anisotropy shows that SA2 binds to single-stranded DNA and DNA with secondary structures (overhang, flap, fork, and replication fork) with higher affinities compared with dsDNA of the same length. Consistent with this observation, AFM imaging shows that SA2 binds to DNA ends and ssDNA gaps with high specificities. It was predicted that DNA ends have a propensity to fray at physiological temperature, which leads to a DNA fork structure with exposed ssDNA (76). Consistent with this theoretical prediction, experiments using 2-aminopurine–substituted DNAs showed enhanced base-flipping activity near DNA ends (77). Consequently, binding of SA2 to DNA ends may be related to its higher binding affinity for DNA with secondary structures, including single-stranded fork. In contrast, the non-ring subunits in the human SMC5/6 complex, hNSE1/3/4, which are also involved in DNA repair and replication, does not exhibit preferential binding to DNA repair and replication–mimicking substrates (78).

SA2 is capable of switching between the diffusive binding (search) mode over dsDNA regions and the stable binding (recognition) mode at the ssDNA gap. Several lines of evidence support that these two DNA-binding modes are reversible, including the following: 1) individual mobile SA2 molecules on gapped DNA tightropes show distinct stable binding events amid free diffusion (Fig. 5*B*); 2) SA2 molecules on gapped DNA tightropes display α-factors < 1 (Fig. 5*A* and Table S1), which indicate pausing amid free diffusion; and 3) sliding window (2 s) MSD analysis of mobile SA2 molecules shows that distinct from the unbiased 1D diffusion mode (∼1.0 × 10^−2^ μm^2^ s^−1^; Fig. S8), on gapped DNA tightropes, mobile SA2 molecules display an additional population with *D*_int_ values centered at ∼1.0 × 10^−4^ μm^2^ s^−1^. Taken together, our results from bulk fluorescence anisotropy and single-molecule experiments strongly suggest that SA2 might play a more prominent role than SA1 at intermediate DNA structures during DNA repair, recombination, and replication.

The crystal structure of human SA2 resembles the shape of an oriental dragon (39, 40). Surface electrostatic potential reveals three positively charged surface patches on SA2 that could be used for nucleic acid binding (Fig. S12). Patch 1 is along the groove located underneath the snout. Patch II is located on the tail of the dragon-shaped molecule and directly faces the back of the dragon head. Another positively charged region, patch III, is found at the backside of the dragon head. A large number of positively charged residues on these three surface patches could potentially mediate interactions with the DNA phosphate backbone. For patch I, the size of the groove under the snout may restrict binding to ssDNA, thus explaining the preference of SA2 for ssDNA over dsDNA molecules. It remains to be determined how much flexibility there is in the overall shape of SA2, as the relative disposition of the dragon head and tail should profoundly impact the affinity of SA2 toward different structured DNA substrates.

Cohesin is required for sister chromatid cohesion at the time of DNA replication or shortly thereafter (79). However, protein-DNA structures that direct the loading of cohesin at the replication fork and the timing of cohesion events relative to the progression of the DNA replication fork are not fully understood (80). Previous studies of cohesin loading onto DNA had been focused on the three ring subunits and their regulators. The results from this study shed new light on a previously uncharacterized function of SA2 in DNA binding. ssDNA gaps and flap structures between Okazaki fragments are generated on the lagging strand after DNA synthesis and displacement of the initial RNA primers (81). Eukaryotic Okazaki fragments (∼150–200 nt) are much shorter than the prokaryotic fragments (∼1200 nt). For every human cell division, >10 million Okazaki fragments are generated. Polymerase δ displacement synthesis on the lagging strand leads to the formation of primarily short flaps, up to 8 nucleotides in length, and a population of flaps up to 20–30 nucleotides. With only slightly weaker affinity for ssDNA in comparison with the single-stranded DNA-binding protein RPA (*K_d_* = 20 nm for 50-nt ssDNA) (82), we expect that SA2 is capable of competing with RPA. ssDNA gap and flap structures on the lagging strand during DNA replication provide ample opportunities for recruiting SA2 during DNA replication. Furthermore, our AFM imaging revealed preferential binding of SA2 at the junction of DNA replication fork (Fig. 8). SA2 can switch between the 1D diffusing (search) mode on dsDNA and stable binding (recognition) mode at ssDNA gaps. Diffusion across dsDNA and ssDNA regions without dissociation would allow individual SA2 molecules to navigate on the lagging strand to form multiprotein SA2 complexes. Recently, single-molecule imaging of QD-labeled *S. pombe* cohesin complexes on DNA curtains suggests that to accommodate both the leading and lagging strands during DNA replication, the bracelet and handcuff models in which each cohesin complex binds to separate DNA strands are most appealing (34). Furthermore, it was shown that the hinge domains SMC1/SMC3 from various organisms contain a basic patch (83). The basic patch in *Bacillus subtilis* SMC (BsSMC) is essential for basal DNA binding by the SMC subunits (84). Importantly, single-molecule fluorescence imaging revealed that BsSMC slides on DNA with diffusion constants consistent with BsSMC making significant contact with DNA during diffusion (85). The findings from this study and previous ones strongly suggest that DNA binding by cohesin is a multistep process involving a composite array of protein-DNA interactions (86).

In addition to DNA replication, cohesin also plays important roles in DNA DSB repair. The observation of preferential binding by SA2 to DNA with secondary structures (overhang, flap, fork, and replication fork) raises another possibility in which SA2 and the cohesin ring bind to separate DNA strands during DNA recombination and repair, perhaps in concert with the MRE11–RAD50–NBS1 (MRN) complex (87). Whereas it has been widely reported that cohesin localizes to dsDNA breaks induced by radiation, enzyme digestion, or DNA replication through DNA lesions (29, 30, 32, 88,89, 90,91), the mechanism underlying cohesin recruitment to regions of dsDNA break is poorly understood. Our observations of HR-mediated DNA DSB repair defect upon knockdown of SA2 are consistent with a previous report showing SA2 recruitment to DSBs and its role in sister chromatid exchange (51). DNA binding by SA2 and its function in HR-mediated DSB repair reported in this study suggest that single-stranded resected DNA, an intermediate structure present during HR, provides the preferred binding site for SA2 and “structure anchor” for the cohesin complex at the DSBs. These results are consistent with a model in which loading of SA2 at the DSBs in coordination with entrapment of its homologous region within the cohesin ring facilitates the HR-mediated DNA DSB repair.

In summary, combining results from this study and a previous one focusing on SA1 (41), we propose that SA1 and SA2 function at specific DNA sequences and structures. The unique roles of SA1 and SA2 are mediated by the difference in their DNA-binding properties. Future studies are needed to identify the DNA-binding domains on SA2 to further define the role of DNA binding by SA2 and other HEAT repeat–containing cohesin and condensin subunits in different DNA maintenance pathways.

## Experimental procedures

### AFM imaging and image analysis

Proteins (60 nm for non-gapped, gapped, and nicked DNA and 160 nm for linear flap, single-stranded fork, replication fork, and circular replication fork DNA) and DNA (2.3 nm for non-gapped, gapped, and nicked DNA and 8 nm for linear flap, single-stranded fork, replication fork, and circular replication fork DNA) were incubated in the SA2-DNA reaction buffer at room temperature for 20 min. The SA2-DNA reaction buffer contains 20 mm Hepes (pH 7.5), 100 mm KCl, and 0.1 mm MgCl_2_. All samples were diluted 10-fold in 1× AFM imaging buffer (25 mm NaOAc, 25 mm HEPES-KOH (pH 7.5), and 10 mm Mg(OAc)_2_) before being deposited onto a freshly cleaved mica surface (SPI Supply). The samples were then washed with MilliQ water and dried under nitrogen gas. All images were collected in the AC mode using MFP-3D-Bio AFM (Asylum Research) and Pointprobe® PPP-FMR probes (Nanosensors, spring constants at ∼2.8 n m^−1^). All images were captured at a scan size of 1–3 μm × 1–3 μm, a scan rate of 1–2 Hz, and a resolution of 512 × 512 pixels. The positions of SA1 and SA2 proteins on DNA were analyzed using software from Asylum Research. DNA-binding specificities (relative affinity of a protein binding to a specific site *versus* a nonspecific site: *S* = *K*_SP_/*K*_NSP_) for DNA ends and ssDNA gaps were calculated based on a method established previously (46),

(Eq. 1) $$S=N\times \frac{A_{\text{SP}}}{A_{\text{NSP}}}+1$$

where *A*_SP_ and *A*_NSP_ are the areas (total number of protein-DNA complexes) in the specific and nonspecific binding regions, respectively, in the protein position distribution histogram. *N* is the number of DNA-binding sites on the linear DNA substrate. The AFM volumes of SA2 complexes were determined using Gwyddion software. Molecular weights of SA2 complexes were estimated based on the calibration curve relating the protein molecular weight (*M*_r_) and AFM volume (*V*, in nm^3^), *V* = 1.45 × *M*_r_ − 21.59 (44).

### Protein-QD conjugation

Biotinylated multivalent chelator ^BT^tris-NTA was prepared according to the previous report (60). For single-color QD labeling of His_6_-tagged WT and mutant SA2 proteins, 0.5 μl of red (655 nm) streptavidin-conjugated QDs (Invitrogen; 1 μm) was incubated with ^BT^tris-NTA (2 μl of 2 μm) for 20 min. Proteins (1 μl of 2 μm) were then added to the QD-NTA solution and incubated in the SA2-DNA reaction buffer for an additional 20 min. For experiments using dual-color labeled QDs, equal molar concentrations of green (565 nm) and red (655 nm) QDs were incubated with ^BT^tris-NTA. For fluorescence imaging, unless otherwise specified, protein-NTA-QD solutions were diluted 200-fold before being introduced into the flow cell (5 nm final protein concentration) in the SA2-DNA reaction buffer using a syringe pump (model SP260p, World Precision Instruments).

### Fluorescence imaging of QD-labeled proteins on DNA tightropes

Fluorescence imaging was carried out with an inverted microscope (Nikon Ti-E) equipped with a solid-state laser (20-milliwatt Sapphire DPSS), a 100× objective with a numerical aperture of 1.49 (APO TIRF; Nikon), and an electron-multiplying CCD camera (iXon DU897, Andor Technology) (57). Construction of the flow cell and formation of DNA tightropes between beads were carried out according to a protocol described previously (56, 57). Polylysine coating enables beads to remain stationary on the PEG-treated coverslip surface during flow stretching of DNA. All data analysis was done using videos collected from using unstained DNA tightropes and under no buffer flow.

The MSD for 1D diffusion as a function of time interval is given by the following,

(Eq. 2) $$\text{MSD}\left(n\Delta t\right)=\frac{1}{N-n}\sum_{i=1}^{N-n}{\left(x_{i+n}-x_{i}\right)}^{2}$$

where *N* is the total number of frames in the trajectory, *n* is the number of frames for different time intervals, Δ*t* is the time between frames, and *x_i_* is the position of the protein-QD on the DNA tightrope in the frame *i.* The 1D diffusion constant (*D*) and α-factor (diffusion exponent) were analyzed by a custom routine developed in LabView based on the following (61).

(Eq. 3) $$\text{MSD}=2Dt^{\alpha }$$

A protein on DNA tightrope was categorized as being mobile if the diffusion constant was >5 × 10^−4^ μm^2^ s^−1^ and the *R*^2^ value from data fitting using Equation 2 was >0.8. To detect static binding events amid 1D diffusion on DNA based on the time interval–based diffusion constant (*D*_int_), we developed a custom MATLAB code to execute “sliding window” (40-frame, 2 s) MSD analysis (41). The custom code is available upon request.

### Fluorescence anisotropy

His_6_-tagged full-length SA2 (amino acids 1–1231, 141 kDa), an SA2 truncation mutant (amino acids 1–1051), or full-length SA1 in the DNA-binding buffer (20 mm Hepes (pH 7.5), 0.1 mm MgCl_2_, 0.5 mm DTT, 100 mm KCl) was titrated into the binding solution containing DNA (1 nm) until the millipolarization stabilized. DNA substrates used in the fluorescence anisotropy are shown Fig. S10A. The data were plotted and analyzed by using the equation, *P* = ((*P*_bound_ − *P*_free_)[protein]/(*K_d_* + [protein])) + *P*_free_, where *P* is the polarization measured at a given total protein concentration, *P*_free_ is the initial polarization of Alexa488-labeled DNA without protein binding, *P*_bound_ is the maximum polarization of DNA due to binding of proteins, and [protein] is the protein concentration.

### DR-GFP reporter assay

The I-SceI–based DR-GFP reporter assay was used to evaluate frequency of DNA DSB repair by homologous recombination as described before (70). DR-GFP–integrated U2OS cells and the pCAGGS vector with I-SceI/GFP were a gift from Dr. Maria Jasin (Memorial Sloan Kettering Cancer Center) and Dr. Jeremy Stark (City of Hope National Medical Center). Briefly, cells were seeded in 24-well plates with reverse transfection of either scrambled control (siCtrl) or SA2 siRNA (siSA2#1, Ambion catalog no. 135923; siSA#2, custom-made from Dharmacon (5′-GUACGGCAAUGUCAAUAUA-3′)). The following day, medium containing the siRNA was then removed, and cells were transfected with I-SceI expression vector along with controls using Lipofectamine 3000. Cells were harvested after 96 h, and GFP-positive cells were quantified using a BD Biosciences flow cytometer. The experiment was performed in triplicate along with appropriate controls (30,000 live cells/sample).

### Statistical analysis

Data from AFM imaging and DNA tightrope assay except for the condition at a lower SA2 concentration (0.6 nm) using λ DNA were pooled from at least 2–3 independent experiments. Unless stated otherwise, the error reported is S.E. Student’s *t* test was used for evaluation of significance in the difference between two sets of measurements. The statistically significant level was set at *p* < 0.05.

## Author contributions

P. C., Y. F., A. G., H. P., E. S., J. L., P. K., X. W., C. W., X. L., N. W., C. Y., and I. T. performed the experiments. P. C. wrote the Matlab code for analysis. I. T., J. P., R. R., A. J. R. B., Y. J. T., and H. W. were involved in the design of the study and writing of the paper.

## Footnotes

2 The abbreviations used are:

DSB: double-strand break
AFM: atomic force microscopy
QD: quantum dot
HR: homologous recombination
nt: nucleotide
NTA: nitrilotriacetic acid
^BT^tris-NTA: biotinylated tris-nitrilotriacetic acid
MSD: mean square displacement.

3 P. Kaur and H. Wang, unpublished data.
