# Supplementary material for: Cohesin SA2 is a sequence-independent DNA-binding protein that recognizes DNA replication and repair intermediates
Source: J Biol Chem. 2020 Aug 27;293(3):1054–69. doi: 10.1074/jbc.M117.806406 (PMC5777247; doi:10.1074/jbc.M117.806406)
Supplement: Supplementary file 1 [file mmc1.zip › jbc.M117.806406-1.pdf]

SUPPLEMENTARY INFORMATION

For manuscript

Cohesin SA2 is a sequence independent DNA binding protein that recognizes DNA replication and repair intermediates

Preston Countryman[1], Yanlin Fan[2], Aparna Gorthi[3,4], Hai Pan[1], Evelyn Strickland[1], Parminder Kaur[1], Xuechun Wang[5], Jiangguo Lin[6,1], Xiaoying Lei[2,7], Christian White[1], Changjiang You[8], Nicolas Wirth[9], , Ingrid Tessmer[9], Jacob Piehler[8], Robert Riehn[1], Alexander J.R. Bishop[3,4], Yizhi Jane Tao[2], Hong Wang[1,10,*]

[1]Physics Department, [5]Biomedical Engineering Department, [10]Center for Human Health and the Environment, North Carolina State University, Raleigh, North Carolina, 27695, USA

[2]Department of BioSciences, Rice University, Houston, Texas, 77251, USA

[3]Greehey Children's Cancer Research Institute, [4]Department of Cell Systems and Anatomy, University of Texas Health at San Antonio, Texas, 78229, USA

[6]Institute of Biomechanics, School of Bioscience and Bioengineering, South China University of Technology, Guangzhou, Guangdong, 510006, P. R. China

[7]School of Public Health, Shandong University, Jinan, 250012, P.R. China

[8]Division of Biophysics, Universität Osnabrück, Barbarstrasse 11, 49076, Osnabrück, Germany

[9]Rudolf Virchow Center for Experimental Biomedicine, University of Würzburg, Josef-Schneider-Str. 2, 97080, Würzburg, Germany

This Supplementary Information contains:

Supplementary Methods
Supplementary Figures S1–12
Supplementary Tables S1-2
Supplementary Legends for Movies S1–3

SUPPLEMENTARY METHODS

Protein purification

The purification of 6xHis tagged full length SA2 (1231 AA, 141 KDa) and a SA2 C-terminal truncation mutant (SA2 1-1051) was described previously (1). Briefly, 6xHis tagged full length SA2 or SA2 1-1051 proteins were overexpressed using a Sf21 insect cell/baculovirus system. Protein purification was done based on affinity purification using Ni-NTA beads (Qiagen) followed by an anion exchange column (Hitrap Q column, GE Healthcare), and a gel filtration column (Superose 6). During purification, SA2 undergoes degradation or proteolytic cleavage by contaminating proteases, resulting in varying amounts of the SA2 (1-1051). The amount of SA2(1-1051) can vary from 0 up to 25% of the total protein for different batches of SA2 (based on SDS-PAGE). The full length SA1 (1258 AA, 141 KDa) containing a N-terminal 3X Flag tag (DYKDHDGDY KDHDIDYKDD DDK) was overexpressed using a baculovirus system and purified using the anti-Flag M2 column (GenScript). mtSSB was provided by the Copeland group at NIEHS.

DNA substrates

The sequences of oligos used to generate DNA substrates for fluorescence anisotropy are shown in Figure S10A. Phage λ DNA was purchased from NEB. pSXneo (T2AG3) plasmid (T270) DNA containing 270 TTAGGG repeats was purchased from Addgene (2). Linearization of T270 DNA was carried out at 37°C for 4 hr using HpaI in the Cutsmart Buffer (NEB).

To generate the plasmid containing centromeric sequences, α-satellite centromeric DNA (1.7 kb) was amplified using a BAC vector containing the centromeric sequences (a gift from Beth Sullivan, Duke University), and subsequently cloned into the pSP73 vector (Promega) using the HpaI restriction site to generate the pSP73C vector. To generate linear DNA substrates with centromeric sequences in the middle (Cen-mid) or close to the end (Cen-end), the pSP73C vector was digested using either ScaI or BglII (NEB). pSCW01 plasmid (duplication of 2030 bp) used for making nicked and gapped DNA substrates was a gift from Peggy Hsieh (NIDDK, NIH) (3). After nicking pSCW01 using Nt.BstNBI (NEB), ssDNA gaps (37 nt) were generated on pSCW01 by repeating the following steps 3 times: introducing complementary oligos (oligos:circular DNA=10:1), heating the sample at 68°C for 30 mins, cooling to room temperature over an hour, and removing extra oligos and short dsDNA using a 100K MW filter (Amicon Ultra) (4). Circular gapped pSCW01 was digested (4 hours, 37 ^{o}C) using either EcoRI in the 3.1 Buffer (NEB) for the DNA tightrope assay or ScaI in the Cutsmart Buffer (NEB) for AFM imaging.

The long linear dsDNA substrates containing flap, single-stranded fork, or replication fork at a defined location were constructed by annealing oligos at the 37-nt ssDNA gap region according to protocols established for making DNA substrates containing mismatches (3). The sequences of the

oligos used for making these substrates are shown in Figure S10C. The Oligo 1 (Figure 10A) was used together with the Fork oligo (Figure S10C) for making the replication fork substrate. Briefly, the Flap, Fork, or Fork plus Oligo 1 (for replication fork) were annealed to the gapped region on circular pSCW01 in the Quick Ligation Reaction Buffer through incubation with oligos at 10-fold excess at 85 ^{o}C for 10 min followed by slow cooling to room temperature. Then the samples were mixed with T4 DNA ligase (NEB) in the presence of ATP to seal the nicks. Ligase was heat denatured by incubating at 65 ^{o}C for 10 mins. Annealing of the oligo at the gapped region was confirmed by restriction digestion using PstI, BamHI, and NcoI for the flap substrate, and by digestion using Pst1 for the single-stranded fork and replication fork substrates in the NEB 1.1 buffer. The circular DNA substrates were linearized using ScaI under the same condition as what used for the gapped DNA, which placed the flap, single-stranded fork, and replication fork at ~23% from one end of the DNA. DNA was purified using the Qiagen PCR DNA purification kit.

The circular DNA substrate containing the replication fork structure was generated using pBluescript KS derived plasmid containing a 398-bp G-less cassette (pGLGAP, a gift from the Griffith group at UNC Chapel Hill) based on a previously established protocol (5). Briefly, the pGLGAP plasmid was nicked using Nb.BbvCI (NEB), followed by nick translation in the presence of dATP, dTTP, and dGTP using the Klenow fragment (exo-, NEB) to generate an ssDNA tail. To create a dsDNA tail, a primer (5' CTTCCTCCATCTATACCACC 3') was annealed to the ssDNA tail, followed by extension using the Klenow fragment (exo-) in the presence of dATP, dTTP, and dCTP. DNA samples were purified using the Zymo DNA Clean & Concentrator™ kit after each enzyme reaction.

To generate the DNA substrate containing a single nick at a defined location, 517 bp PCR fragments obtained using pUC18 as the template (1370 to 1887) was nicked using Nt.BstNBI. Nicking of the 517 bp DNA was confirmed by electrophoresis (6).

To generate longer DNA substrates for fluorescence imaging of QD-labeled proteins on DNA tightropes, linearized DNA fragments (telomeric, centromeric, or genomic) were ligated using a Quick Ligation™ Kit (NEB). The ligation reactions were incubated at room temperature for 1 hr for pSCW01 derived DNA substrates and 15 min for all other DNA substrates. All ligated DNA substrates were further purified using phenol-chloroform extraction. DNA tightropes with ssDNA gaps were generated by introducing complementary oligos inside flow cells, heating flow cells at 55°C for 90 mins, and washing flow cells with 300 μl of $MgCl_2$ (1 M), followed by 3 ml of H_2O to remove extra oligos and short dsDNA.

Table S1: Dynamics of QD-labeled full length SA2 on different DNA tightropes.

DNA	WT SA2-QDs (5 nM) D ($\mu m^2\ s^{-1}$)	WT SA2-QDs (5 nM) Alpha factor	WT SA2-QDs (0.6 nM) D ($\mu m^2\ s^{-1}$)	WT SA2-QDs (0.6 nM) Alpha factor	WT SA2-QDs (0.6 nM) Static (%)
Centromeric	0.10±0.02 (N=48)	0.96±0.02 (N=65)	-	-	24
T270	0.10±0.02 (N=53)	0.86±0.03 (N=55)	-	-	46
λ DNA	0.09±0.02 (N=48)	0.93±0.04 (N=48)	0.13±0.03 (N=19)	0.96±0.03 (N=19)	-
Nicked λ DNA	-	-	0.08±0.03 (N=20)	0.94±0.04 (N=20)	-
Gapped DNA	-	-	0.01±0.003 (N=28)	0.70±0.05 (N=28)	81

Note: D: diffusion constant (mean±SEM)

Table S2: The equilibrium dissociation constants (K_d) of the full length SA2 for different DNA substrates measured from fluorescence anisotropy experiments.

DNA substrates		K_d [nM] (mean±SEM)
**dsDNA non-telomeric**	**66 bp**	76.2 ± 3.9
	**45 bp**	175.3 ± 12.9
	**25 bp**	ND
**dsDNA telomeric**	**66 bp**	88.0 ± 1.5
**ssDNA**	**66 nt**	41.0 ± 5.3
	**45 nt**	117.6 ± 5.3
	**25 nt**	445.2 ± 11.9*
	**15 nt**	1003.4 ± 0.5*
**overhang**		56.4 ± 9.0
**fork**		58.4 ± 9.4
**flap**		103.8 ± 11.7
**replication fork**		132.7 ± 33.5

*indicates uncertainty in the determination of the equilibrium dissociation constant (K_d) due to the low percentage of DNA binding at the highest final SA2 concentrations tested. K_d was calculated from two independent experiments. Sequences of DNA substrates are shown in Figure S10A.

Movie S1. A SA2-QD complex displaying unbiased 1D diffusion on a centromeric DNA tightrope. The scale bar is 1 μm. Original 2-min movie playing at 2X speed. The left and right panels show the movie and corresponding kymograph, respectively, of green QD-labeled His-tagged full length SA2 on the centromeric DNA tightrope.

Movie S2. A SA2-QD complex displaying alternation between unbiased 1D diffusion and stable binding on a gapped DNA tightrope. The scale bar is 1 μm. Original 2-min movie playing at 2X speed. The left and right panels show the movie and corresponding kymograph, respectively, of QD-labeled His-tagged full length SA2 on the gapped DNA tightrope. His-tagged SA2 was incubated with ^{BT}tris-NTA and equal molar concentrations of green and red streptavidin-conjugated QDs.

Movie S3. SA2 bypassing diffusion barriers posed by another DNA-bound SA2 molecule on λ DNA. The scale bar is 1 μm. Original 2-min movie playing at 2X speed. The left and right panels show the movie and corresponding kymograph, respectively, of QD-labeled His-tagged full length SA2 on a λ DNA tightrope. His-tagged SA2 was incubated with ^{BT}tris-NTA and equal molar concentrations of green and red streptavidin-conjugated QDs.

SUPPLEMENTARY REFERENCES

1. Zhang, N., Jiang, Y., Mao, Q., Demeler, B., Tao, Y.J. and Pati, D. (2013) Characterization of the interaction between the cohesin subunits Rad21 and SA1/2. *PloS one*, **8**, e69458.
2. Hanish, J.P., Yanowitz, J.L. and de Lange, T. (1994) Stringent sequence requirements for the formation of human telomeres. *Proceedings of the National Academy of Sciences of the United States of America*, **91**, 8861-8865.
3. Geng, H., Du, C., Chen, S., Salerno, V., Manfredi, C. and Hsieh, P. (2011) In vitro studies of DNA mismatch repair proteins. *Analytical biochemistry*, **413**, 179-184.
4. Buechner, C.N. and Tessmer, I. (2013) DNA substrate preparation for atomic force microscopy studies of protein-DNA interactions. *Journal of molecular recognition : JMR*, **26**, 605-617.
5. Subramanian, D. and Griffith, J.D. (2005) p53 Monitors replication fork regression by binding to "chickenfoot" intermediates. *The Journal of biological chemistry*, **280**, 42568-42572.
6. Wang, H., Tessmer, I., Croteau, D.L., Erie, D.A. and Van Houten, B. (2008) Functional characterization and atomic force microscopy of a DNA repair protein conjugated to a quantum dot. *Nano letters*, **8**, 1631-1637.

A B

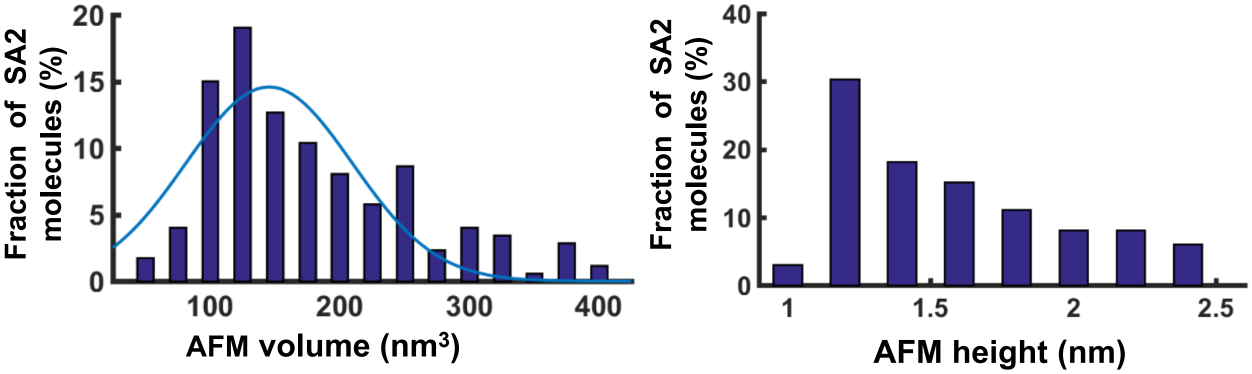

C

DNA substrates	At DNA ends (mean±SD) (nm³)	At non-gapped regions (mean±SD) (nm³)	At ssDNA gaps (mean±SD) (nm³)
**Non-gapped DNA**	1025±88 (N=34)	898±63 (N=68)	-
**Gapped DNA**	786±98 (N=25)	1096±117 (N=45)	1458±232 (N=26)

Figure S1. Evaluation of the oligomeric state of SA2 in solution and on DNA. (**A**) The AFM volume of SA2 alone in solution. SA2 volume was measured using the Gwyddion software. The solid line is the Gaussian fit to the data (N=173) with the peak centered at 146 nm^3. The expected AFM volume of full length SA2 monomer (141 KDa) is ~180 nm^3 based on the calibration curve V = 1.45 Mr – 21.59, where V is the AFM volume (nm^3) and Mr is the molecular weight (KDa) of the protein (Kaur et al. 2016). (**B**) SA2 AFM height distribution on the linear gapped DNA substrate (N=173). (**C**) Comparison of AFM volumes of SA2 on the linear non-gapped and gapped DNA substrates. The estimation of the number of SA2 molecule is based on the calibration curve relating the protein molecular weight and its AFM volume (V = 1.45 MW – 21.59).

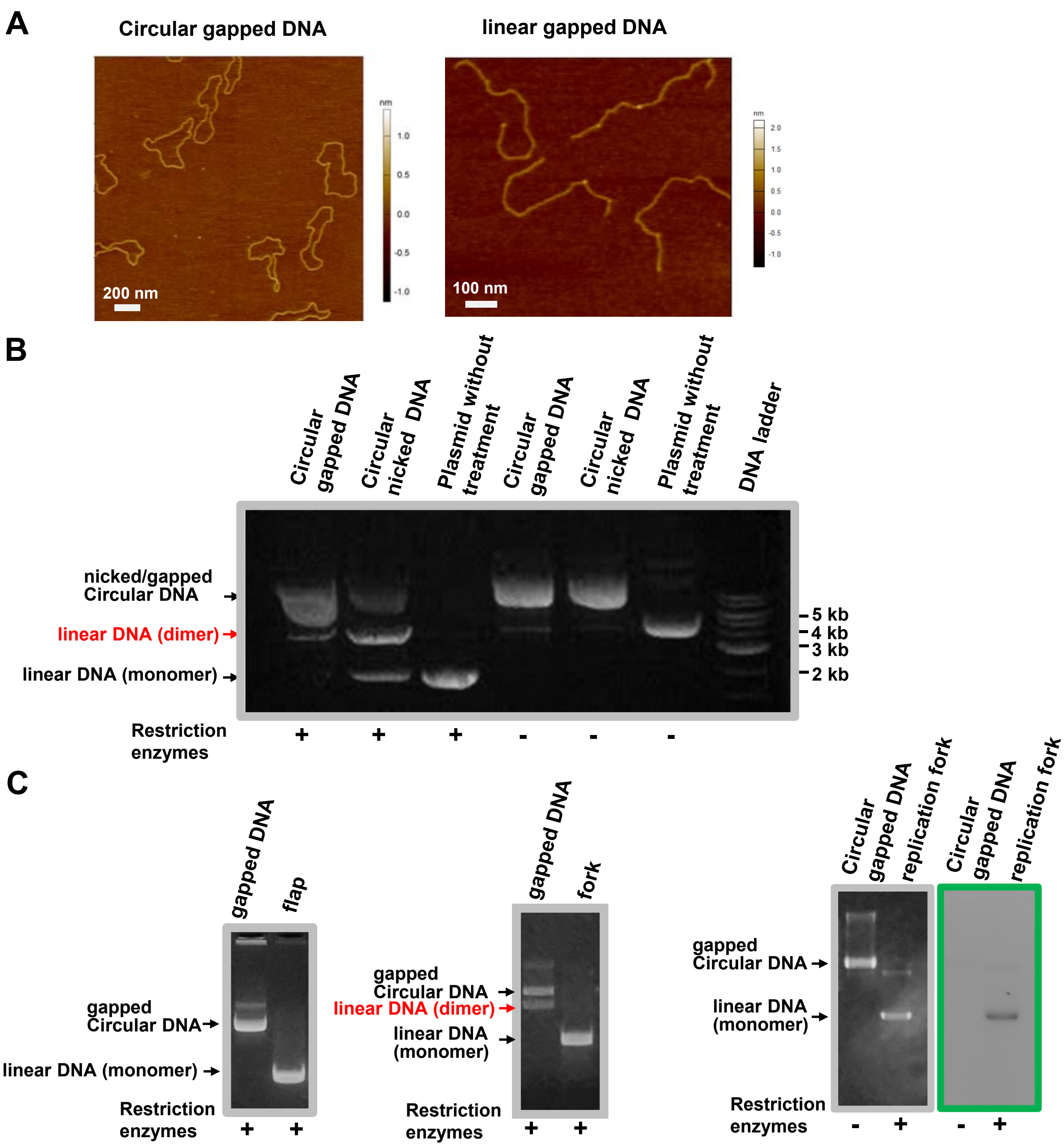

Figure S2. Characterization of the DNA substrates containing ssDNA gap, flap, single-stranded fork, and replication fork. (**A**) AFM images of the circular gapped (left panel) and linearized gapped DNA substrates. Circular DNA was digested with ScaI to generate the linear gapped DNA substrate with the ssDNA gap at 470 bp from one end. Circular gapped DNA substrate exists as dimers and displays contour length (1354.0±29.0 nm, mean±SD) that is approximately twice that of the linearized fragment (622.5±41.3 nm, mean±SD). (**B**) Quantification of the DNA gapping efficiency using restriction digestion. Circular DNA was digested with NcoI, BamHI and PstI, which are located between nicked sites. The percentages of digestion were approximately 60% and 5%, respectively, for nicked and gapped DNA substrates. (**C**) Validation of the flap, single-stranded fork, and replication fork DNA substrates by restriction analysis. Incorporation of oligos at the ssDNA gap regions enabled restriction digestion. Incorporation of the second Alexa488 labeled oligo (Figure S10) complementary to the single-stranded fork region was confirmed by scanning the gel using a Typhoon scanner (right panel). Note that gapping efficiencies varied from batch to batch (compare digestion of gapped DNA in B and C). Gray boxes mark gel pictures taken under UV with DNA stained with SafeStain, and the green box marks the gel picture taken on a Typhoon scanner).

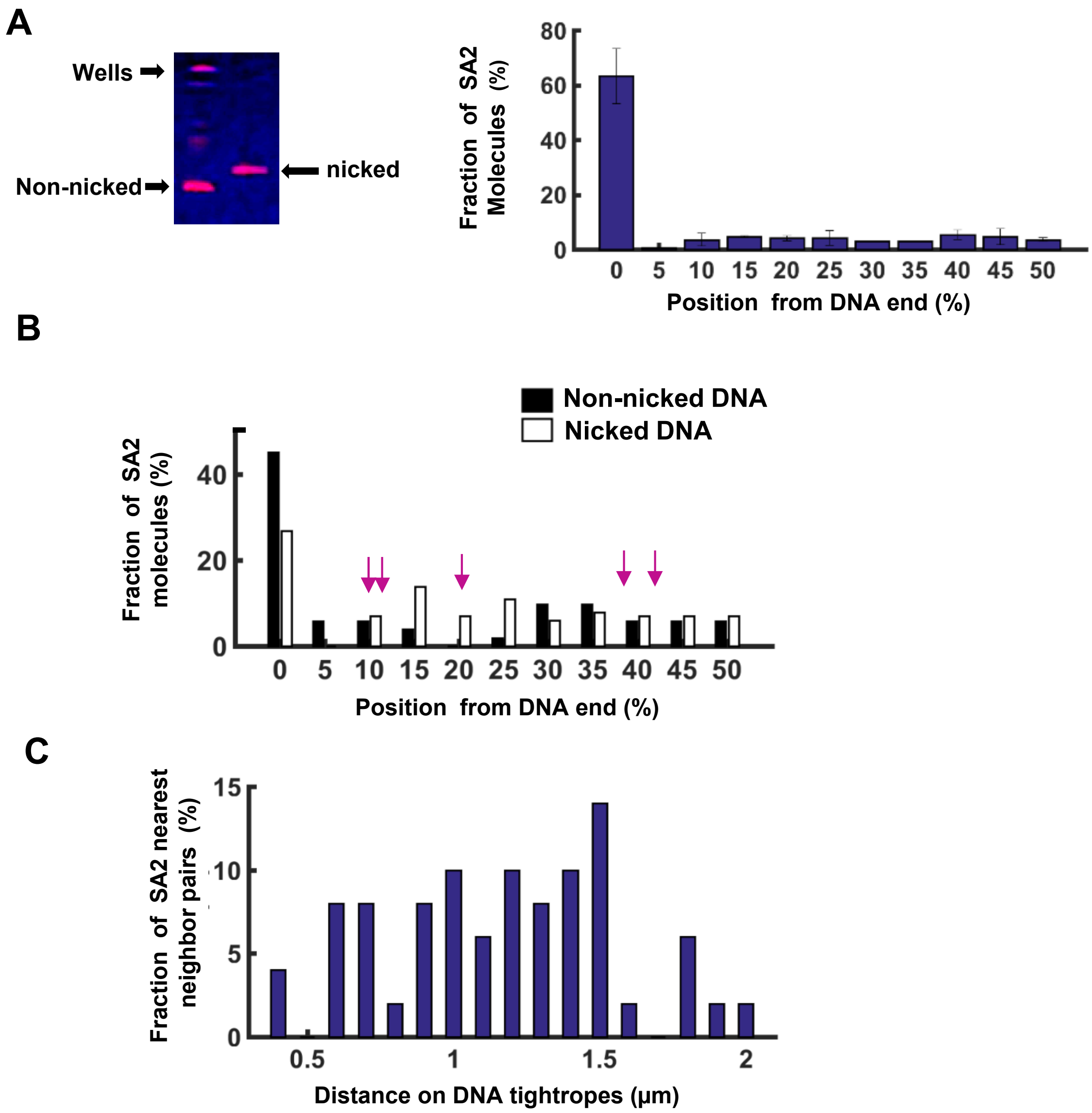

Figure S3. Binding locations of SA2 on DNA substrates containing either a single or multiple nick sites are random. (**A**) Characterization of linear DNA fragment (517 bp) containing a single nick at 40% from one end (left panel) and binding positions of SA2 on this DNA substrate (N=169, right panel) observed in AFM images. The error bars represent SEM. Generation of this linear nicked DNA substrate is described in Wang et al., 2008. (**B**) Positions of SA2 on linear DNA substrates before (N=51, black bars) and after (N=101, white bars) treatment with nickase observed in AFM images. The linear nicked DNA substrate (pUC19 derived) contains a total of 5 nicks: at ~10% (2 nicks), 20%, 38%, and 43% from one end (purple arrows). (**C**) Spacing between nearest neighbor pairs of SA2-QDs (N=71) on nicked pSCW01 DNA tightropes is random.

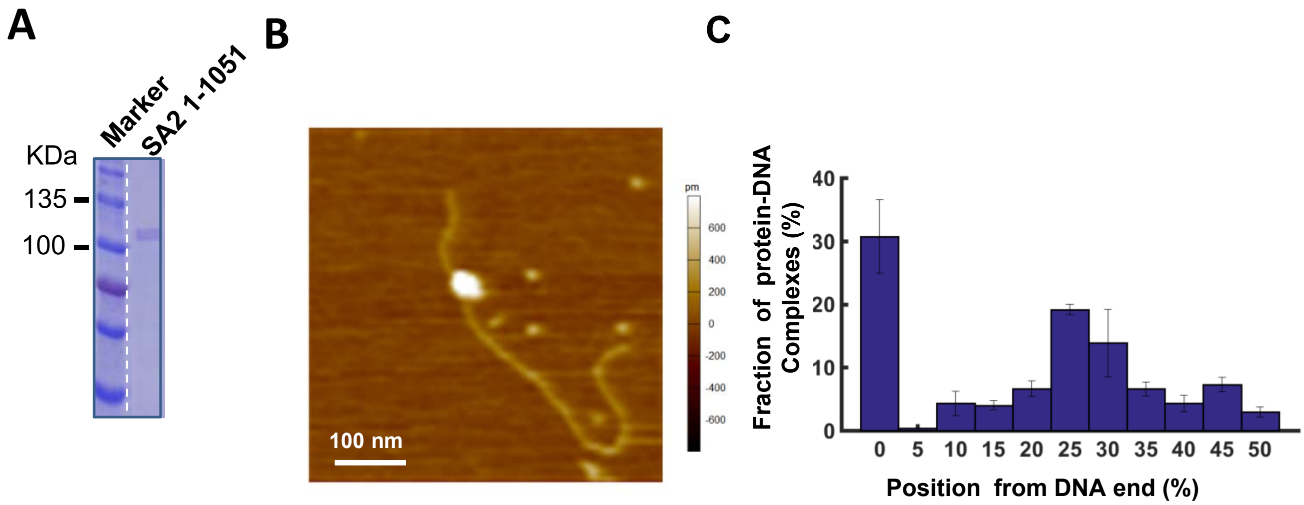

Figure S4. The C-terminal deletion SA2 mutant (SA2 1-1051 AA) retains binding specificities for DNA ends and ssDNA gaps. (**A**) SDS-PAGE gel of purified SA2 1-1051. (**B**) An AFM image of SA2 1-1051 binding to the linear gapped DNA substrate. (**C**) Statistical analysis of the position distribution of SA2 1-1051 complexes on the gapped DNA (N=303) in AFM images. The data are from three independent experiments. The error bars represent SEM.

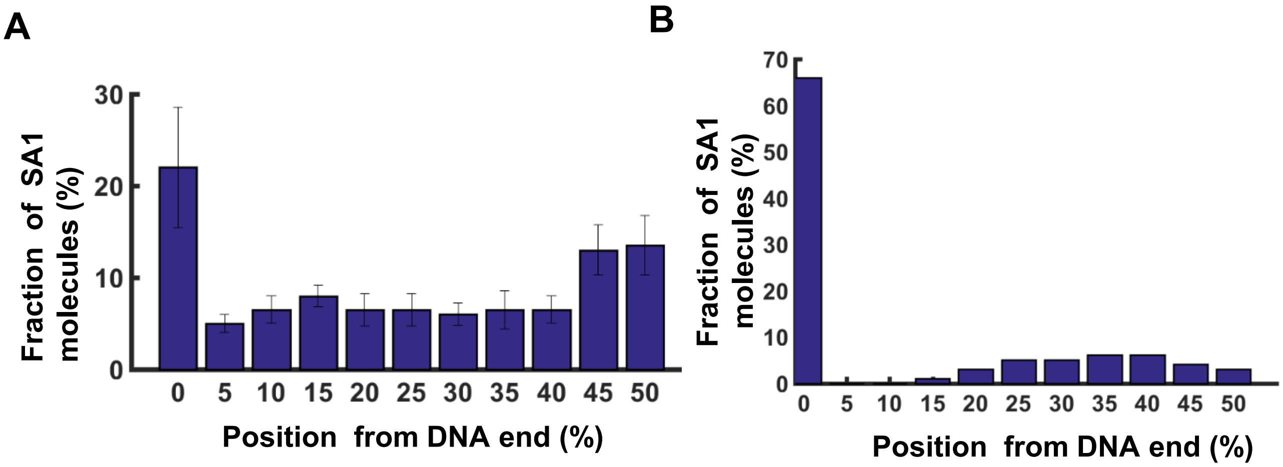

Figure S5. Full length SA1 recognizes DNA ends, but does not show preferential binding to DNA nicks. (**A** and **B**) Analysis of the binding position of SA1 on the telomeric T270 DNA substrate including DNA end binding (**A**, N=200), and SA1 on the linear DNA substrate (517 bp) containing a single nick (**B**, N=97) in AFM images.

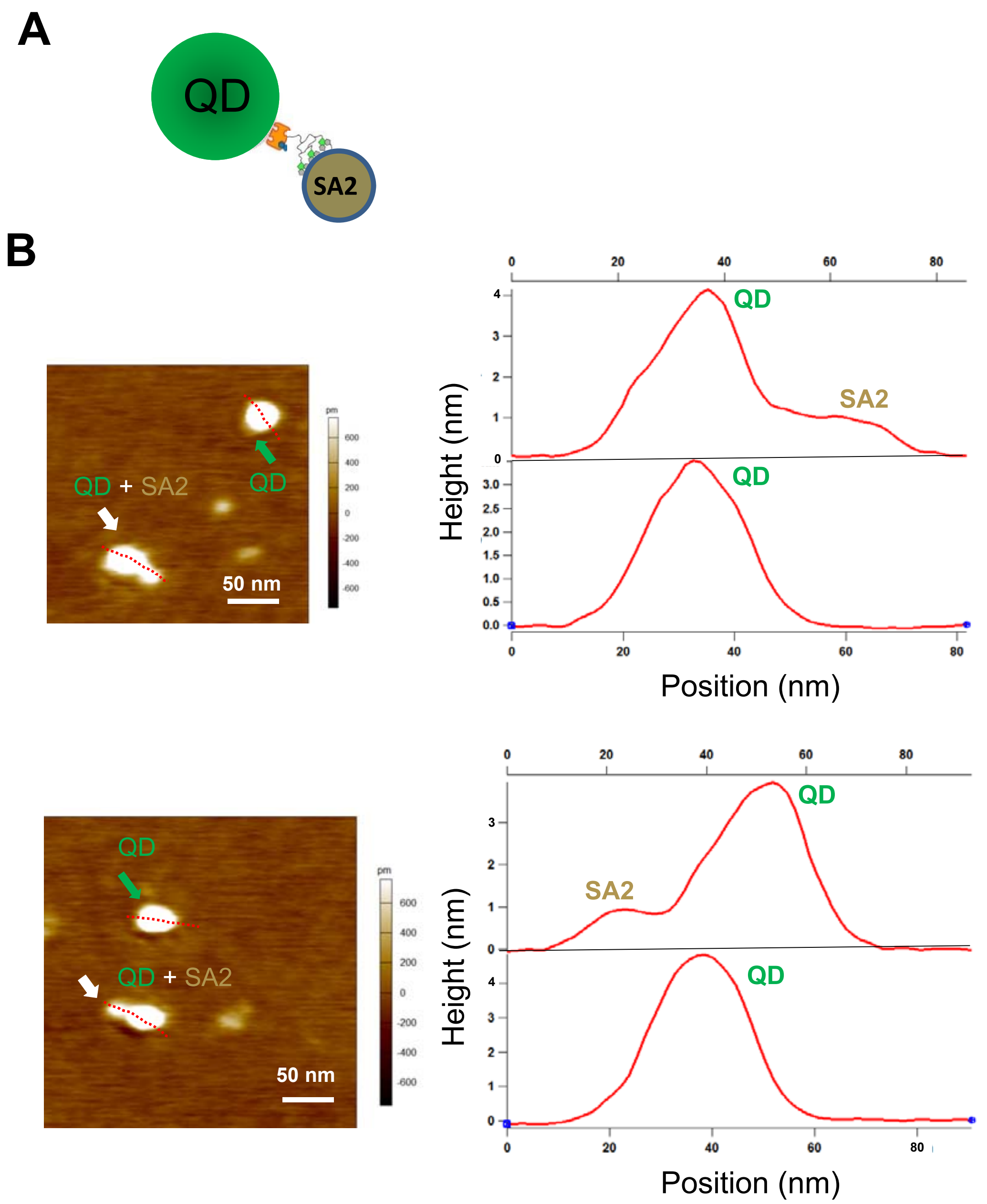

Figure S6. Characterization of stoichiometry of SA2-QDs by AFM imaging. (**A**) A schematic representation of His-tagged SA2 conjugated to a green (565 nm) streptavidin-coated QD through the ^{BT}tris-NTA compound. Commercially available streptavidin-coated QDs contain multiple of streptavidin molecules on individual QDs. (**B**) AFM images (left panels) and cross section analysis (right panels) of streptavidin-coated QDs in the presence of SA2 (SA2:QD=4:1). Green and white arrows point to QD alone and SA2-QDs with a single SA2 molecule, respectively. The section analysis on the right is from the path drawn in the AFM images on the left (red line). The heights of SA2 and QDs in the SA2-QD conjugates (N=116) are 1.0 ± 0.2 nm and 3.7 ± 0.7 nm, respectively. Among the QDs (N=407) showing SA2 attached in AFM images, 101 were labeled with a single SA2 molecule, while 11 comprise of SA2-QDs with 2 SA2 molecules. Only 4 SA2-QDs show three SA2 molecules attached.

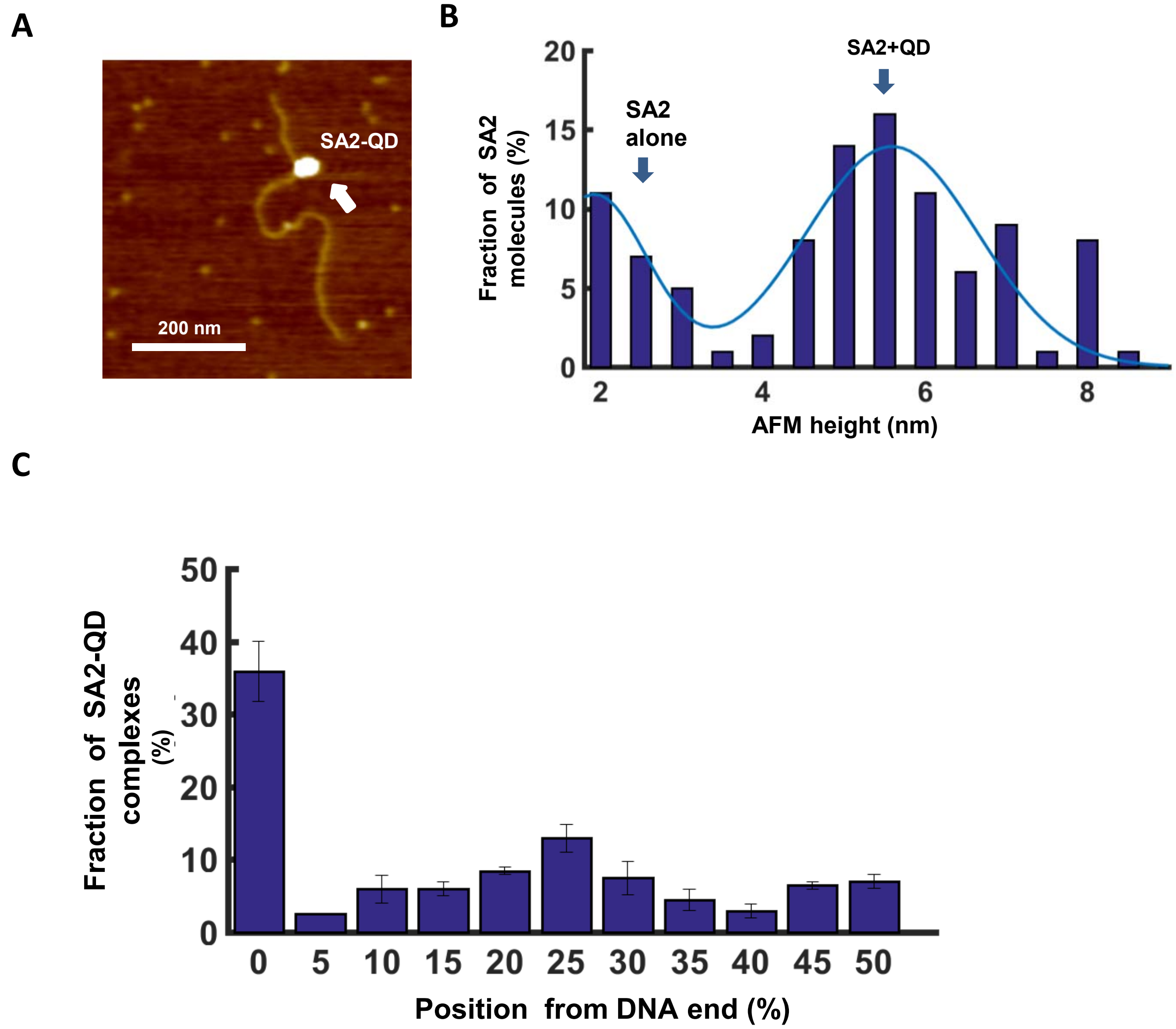

Figure S7. QD-labeled SA2 retains specific binding to DNA ends and ssDNA gaps. (**A**) An AFM image of QD-labeled full length SA2 on the linear gapped DNA. The white arrow points to a SA2-QD complex binding to a location consistent with an ssDNA gap (23% from one end, Figure 3A). (**B**) Statistical analysis of the AFM height of complexes (N=109) formed on gapped DNA when both SA2 and QDs were present in solution. The solid line shows the Gaussian fit (R^2 >0.72) with the peaks centered at 1.9±1.3 nm and 5.6 ± 0.2 nm, respectively. (**C**) The position distribution of SA2-QDs on the gapped DNA substrate (N=201). SA2-QDs were identified as complexes with heights greater than 3.5 nm. The error bars represent SEM.

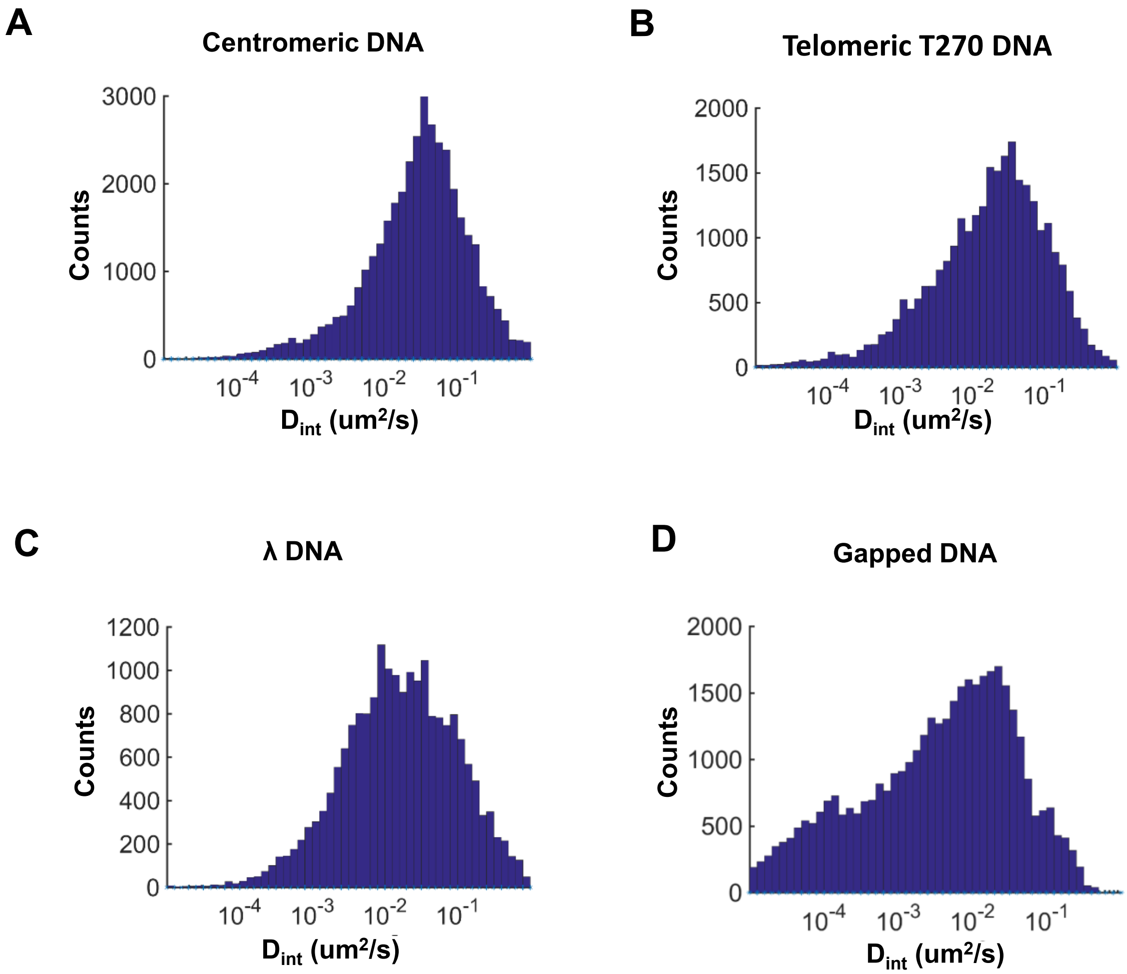

Figure S8. Comparison of the distribution of interval based diffusion constants (D_{int}) for SA2 on different DNA tightropes. D_{int} distributions for all mobile SA2 molecules observed on centromeric (**A**, N=48 SA2 molecules), telomeric (**B**, N=52 SA2 molecules), λ (**C**, N=41 SA2 molecules), and gapped DNA (**D**, N=31 SA2 molecules).

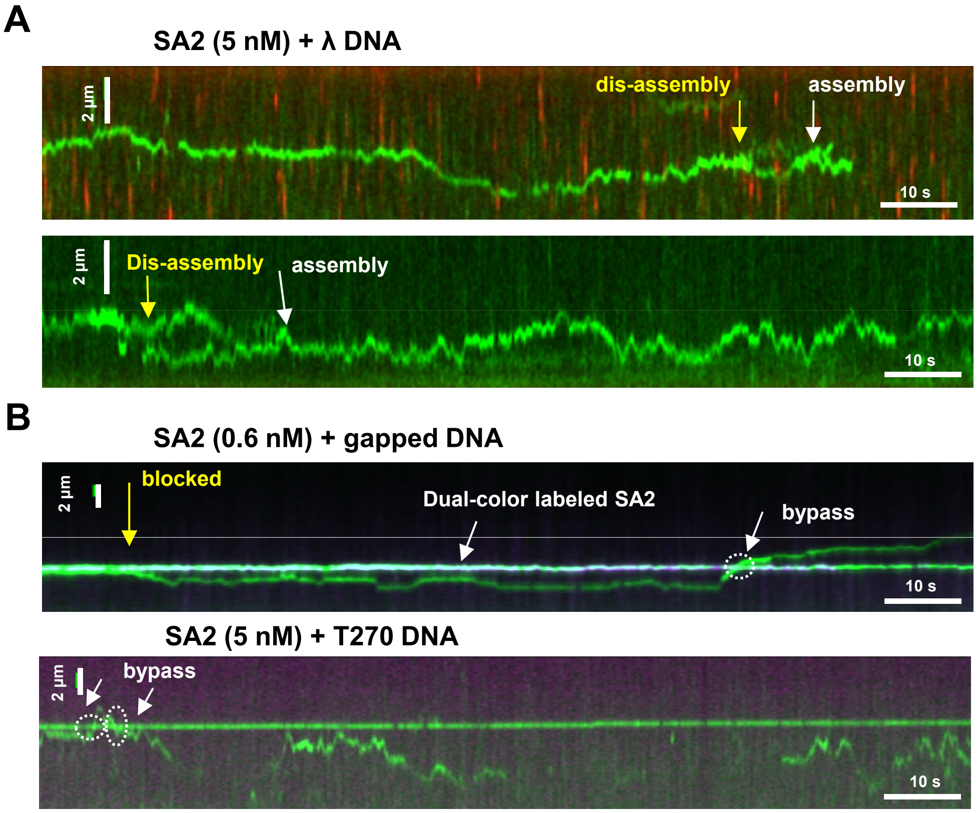

Figure S9. SA2 forms dynamic higher-order oligomeric complexes on DNA and can bypass another DNA-bound SA2 complex. (**A**) Kymographs of SA2-QDs on DNA showing dis-assembly (yellow arrows) and assembly (white arrows) of SA2 complexes on λ DNA tightropes. (**B**) Examples of SA2 bypassing diffusion barriers posed by another DNA-bound SA2 molecules on gapped (top panel) and telomeric (T270, bottom panel) DNA tightropes. Yellow arrow: SA2 was blocked by another DNA-bound SA2-QD, white circles: the bypass events.

A

Alexa ss66:
5'CTGGATCCGTACAGTGTAAGGGTGAGTGGTTGGGTGTGTGGGTGTTGATGTAGAACAAGAATTCGA3'
ss66-com:
5' TCGAATTCTTGTTCTACATCAACACCCACACACCCAACCACTCACCCTTACACTGTACGGATCCAG3'
Oligo 1 (Alexa ss45): 5' Alexa 488 TGTCGCATAGTGTAGTCGGTCTTGTTCGGTCATAGCTCATCGTGG3'
Oligo 2: 5'CCACGATGAGCTATGACCGAACAAGACCGACTACACTATGCGACA3
Oligo 3:5'CCACGATGAGCTATGACCGAACAA3'
Oligo 4: 5'CCACGATGAGCTATGACCGAATGCCGAATTCTACCAGTGCCAGTG3'
Oligo 5: 5'ACTCTGTTCATCGTCATCTGCGACCGACTACACTATGCGACA3'
Oligo 6: 5'GCAGATGACGATGAACAGAGT3'
Oligo 7: 5'CACTGGCACTGGTAGAATTCGGCAGCAGATGACGATGAACAGAGT3'
Alexa ss25: 5'AlexA 488 TGTCGCATAGTGTAGTCGGTCTTGT3'
Alexa ss15: 5'Alexa 488 TGTCGCATAGTGTAG3'
ss-25-com: 5'ACA AGA CCG ACT ACA CTA TGC GAC A3'

B

DNA substrates for fluorescence anisotropy

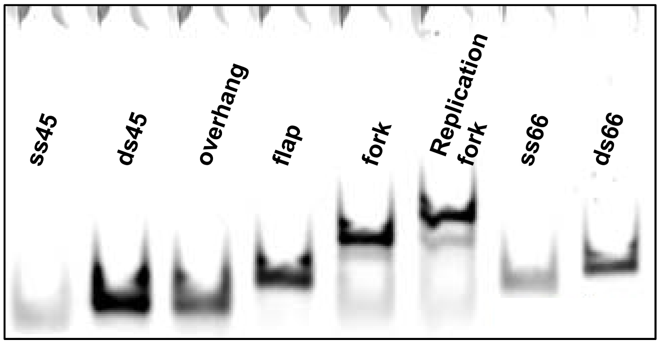

C

Oligos for making DNA substrates for AFM imaging

Flap oligo
5' **actctgttcatcgtcatctgc** CCG AGT CAT TCC TGC AGC GAG TCC ATG GGA GTC AAA T 3'

Fork oligo
5' phos/CCG AGT CAT TCC TGC AGC GAG TCC ATG
CCACGATGAGCTATGACCGAACAAGACCGACTACACTATGCGACA 3'

Figure S10. DNA substrates used for fluorescence anisotropy. (**A**) Sequences of oligonucleotides for generating DNA substrates (Figure 6B) for testing SA2 binding to ssDNA (66, 45, 25, and 15 nt), dsDNA (66, 45, and 25 bp), and DNA with secondary structures (overhang, flap, fork, and replication fork). (**B**) Native polyacrylamide gel electrophoresis showing annealed products and their ssDNA counterparts. ds45 (oligo 1 + oligo 2); ds66 (Alexa ss66 + ss-66-com). The schematic illustration of DNA substrates is shown in Figure 6A. (**C**) Sequences of the oligos used for making the flap, single-stranded fork, and replication fork substrates. The sequences underlined are complementary to the ssDNA gap region. The replication fork substrates were made by adding both the Fork oligo and Oligo 1 during the annealing process. The sequences in purple indicate the region on the Fork oligo that is complementary to Oligo 1.

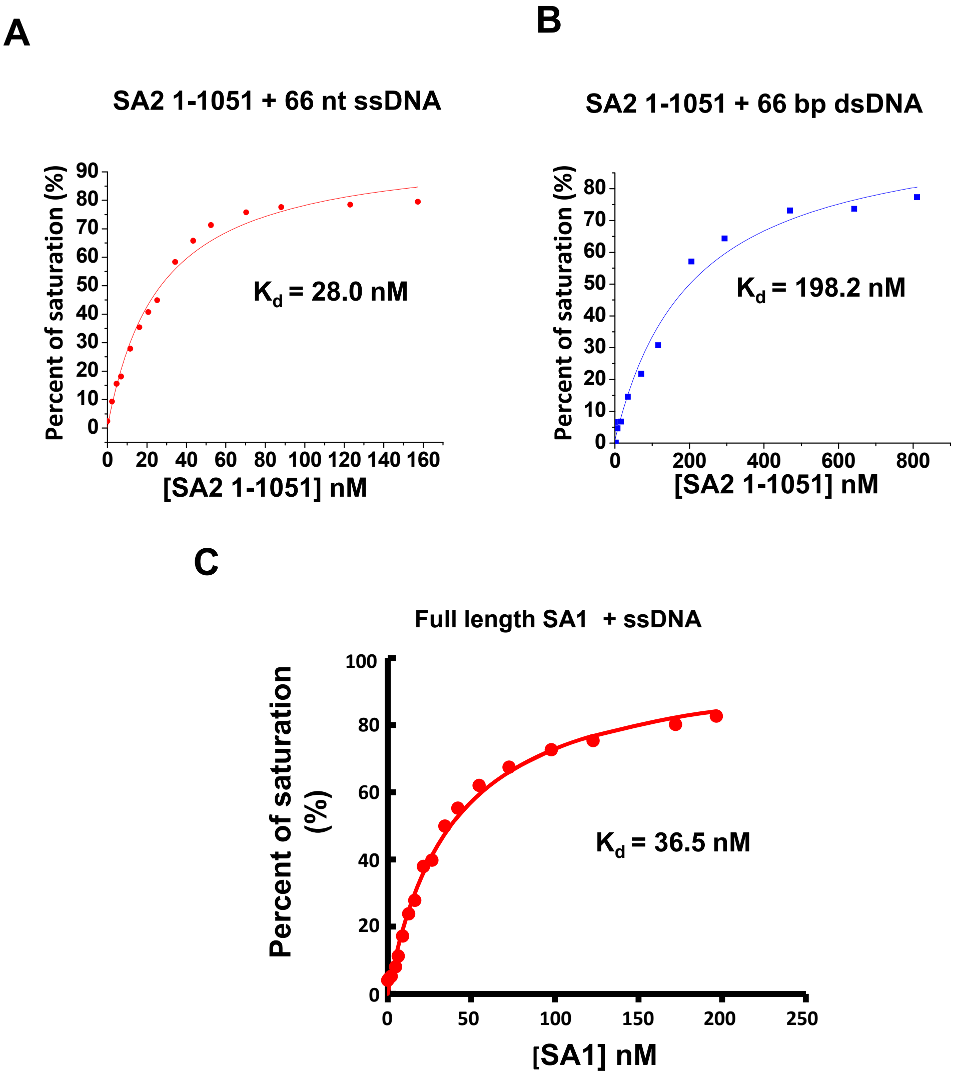

Figure S11. DNA binding by SA2 1-1051 and SA1. (**A-B**) Fluorescence anisotropy experiments showing concentration-dependent binding of SA2 1-1051 to the single-stranded (Alexa ss66 in Figure S9A, **A**) and double-stranded DNA substrates (Alexa ss66/ss66-com, **B**). The data were fitted to the law of mass action. DNA substrates are labeled with Alexa 488. The equilibrium dissociation constants are 28.0 and 198.2 nM, respectively, for binding of SA2 1-1051 to ss (two independent experiments) and dsDNA. (**C**) Fluorescence anisotropy experiments showing concentration-dependent binding of SA1 to the Alexa 488-labeled ssDNA substrate (Alexa ss66 in Figure S9A). The data were fitted to the law of mass action. The equilibrium dissociation constant is 36.5±0.2 nM (two independent experiments) for SA1 binding to ssDNA (66 nt).

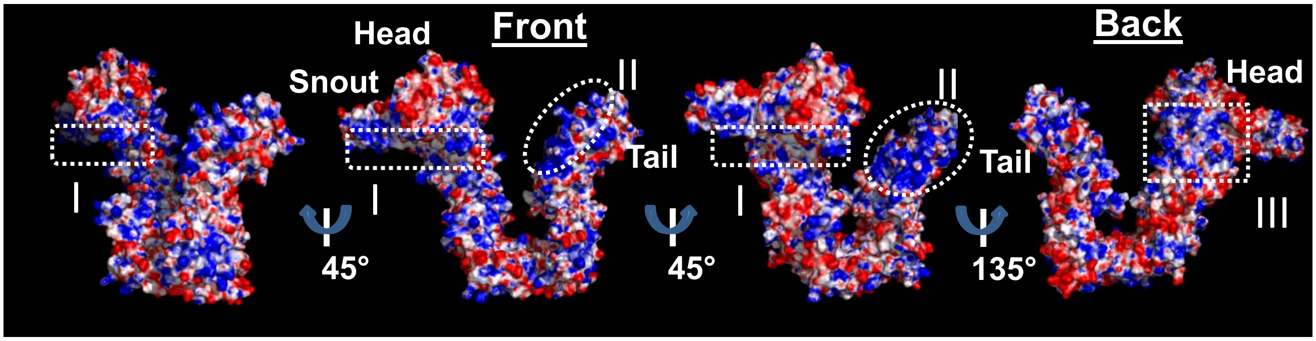

Figure S12. Potential DNA binding sites on SA2. Molecular surface of human SA2 (AA 80—1060), colored by electrostatic potential with positive in blue and negative in red, is viewed from different angles. The 2nd and 4th panels represent the front and back view of the dragon-shaped SA2 molecule, respectively. Three positively charged surface patches I (rectangle with round corners), II (ovals) and III (rectangle with square corners) are highlighted by dashed objects. The figures were created using PBD 4PJU (Hara et al., 2014) and PyMOL software (The PyMOL Molecular Graphics System, Version 2.0 Schrödinger, LLC).
